# Plant resistance inducer AMHA enhances antioxidant capacities to promote cold tolerance by regulating the upgrade of glutathione S-transferase in tea plant

**DOI:** 10.1093/hr/uhaf073

**Published:** 2025-03-05

**Authors:** Xuejin Chen, Ning Zhou, Lisha Yu, Zhaolan Han, Yanjing Guo, Salome Njeri Ndombi, Huan Zhang, Jie Jiang, Yu Duan, Zhongwei Zou, Yuanchun Ma, Xujun Zhu, Shiguo Chen, Wanping Fang

**Affiliations:** Tea Science Research Institute, Weed Research Laboratory, Binjiang Campus, Nanjing Agricultural University, No. 555, Binjiang Avenue, Pukou District, Nanjing, Jiangsu Province, China; Tea Science Research Institute, Weed Research Laboratory, Binjiang Campus, Nanjing Agricultural University, No. 555, Binjiang Avenue, Pukou District, Nanjing, Jiangsu Province, China; Tea Science Research Institute, Weed Research Laboratory, Binjiang Campus, Nanjing Agricultural University, No. 555, Binjiang Avenue, Pukou District, Nanjing, Jiangsu Province, China; Tea Science Research Institute, Weed Research Laboratory, Binjiang Campus, Nanjing Agricultural University, No. 555, Binjiang Avenue, Pukou District, Nanjing, Jiangsu Province, China; Tea Science Research Institute, Weed Research Laboratory, Binjiang Campus, Nanjing Agricultural University, No. 555, Binjiang Avenue, Pukou District, Nanjing, Jiangsu Province, China; Tea Science Research Institute, Weed Research Laboratory, Binjiang Campus, Nanjing Agricultural University, No. 555, Binjiang Avenue, Pukou District, Nanjing, Jiangsu Province, China; Tea Science Research Institute, Weed Research Laboratory, Binjiang Campus, Nanjing Agricultural University, No. 555, Binjiang Avenue, Pukou District, Nanjing, Jiangsu Province, China; Tea Science Research Institute, Weed Research Laboratory, Binjiang Campus, Nanjing Agricultural University, No. 555, Binjiang Avenue, Pukou District, Nanjing, Jiangsu Province, China; Tea Science Research Institute, Weed Research Laboratory, Binjiang Campus, Nanjing Agricultural University, No. 555, Binjiang Avenue, Pukou District, Nanjing, Jiangsu Province, China; Department of Biology, Wilfrid Laurier University, 75 University Avenue West, Waterloo, ON N2L 3C5, Canada; Tea Science Research Institute, Weed Research Laboratory, Binjiang Campus, Nanjing Agricultural University, No. 555, Binjiang Avenue, Pukou District, Nanjing, Jiangsu Province, China; Tea Science Research Institute, Weed Research Laboratory, Binjiang Campus, Nanjing Agricultural University, No. 555, Binjiang Avenue, Pukou District, Nanjing, Jiangsu Province, China; Tea Science Research Institute, Weed Research Laboratory, Binjiang Campus, Nanjing Agricultural University, No. 555, Binjiang Avenue, Pukou District, Nanjing, Jiangsu Province, China; Tea Science Research Institute, Weed Research Laboratory, Binjiang Campus, Nanjing Agricultural University, No. 555, Binjiang Avenue, Pukou District, Nanjing, Jiangsu Province, China

## Abstract

Plant resistance inducers represent an alternative strategy that mitigate stress-induced damage in plants. Previously, 2-amino-3-methylhexanoic acid (AMHA), a novel natural plant resistance inducer, was shown to significantly bolster cold tolerance, thermotolerance, and pathogen resistance in plants. However, the intricate mechanisms underlying AMHA’s response to cold stress remain elusive. Thus, we investigated the physiological and transcriptomic analyses of AMHA pretreatment on tea plant to determine its substantial role of AMHA under cold stress. The results showed that pretreatment with 100 nM AMHA effectively mitigated the detrimental effects of cold stress on photosynthesis and growth. Furthermore, differentially expressed genes were identified through RNA-seq during pretreatment, cold stress, and 2 days of recovery. These genes were mainly enriched in pathways related to flavonoid/anthocyanin, carotenoid, and ascorbic acid-glutathione (AsA-GSH) cycle, including *GST* (encoding glutathione S-transferase). Potential regulatory relationships between the identified genes and transcription factors were also established. Antisense oligodeoxynucleotide-silencing and overexpression experiments revealed that *CsGSTU7* enhances cold resistance by maintaining redox homeostasis. In conclusion, our study suggests that antioxidant-related signaling molecules play a critical role in the signaling cascades and transcriptional regulation mediating AMHA-induced cold-stress resistance in tea plant.

## Introduction

Cold stress represents a formidable environmental challenge that profoundly influences plant growth, geographical distribution, phenological development, and productivity [1]. It elicits a cascade of detrimental effects on various physio-biochemical processes within plants, notably including the precocious closure of stomata, suppression of photosynthesis, augmentation of reactive oxygen species (ROS) toxicity, and degradation of cell membranes. These disruptions are primarily attributed to impairments in gene transcription and signal transduction in plants [2]. Notably, the photosynthetic apparatus, specifically photosystem I (PSI) and photosystem II (PSII), which serve as the cornerstone of electron transport in plants, is particularly susceptible to cold stress [3]. Cold stress disrupts the delicate balance of these photosystems, impeding electron transport and reducing the activity of carbon assimilation enzymes, thereby leading to a decline in photosynthetic efficiency and an accumulation of ROS [4]. As sessile organisms, plants have evolved intricate systems to mitigate the adverse effects of cold stress. A pivotal component of these defenses is the antioxidative defense system, encompassing both enzymatic and non-enzymatic components. Key enzymes, such as peroxidase (POD), catalase (CAT), superoxide dismutase (SOD), ascorbate peroxidase (APX), and glutathione peroxidase (GPX), play a vital role in scavenging excessive ROS production and maintaining redox homeostasis [5]. Furthermore, ascorbic acid (AsA) and glutathione (GSH) function as primary antioxidants, mitigating ROS-induced cellular damage [6]. Cold stress also affects various metabolic pathways, such as those involved in anthocyanins, carotenoids, and soluble sugars production, causing oxidative damage [7]. Therefore, elevating the antioxidant activities of plants is crucial for enhancing their resistance to cold stress.

The tea plant (*Camellia sinensis* L. O. Kuntze) is an economically crucial beverage crop that is susceptible to a wide range of environmental stresses [8, 9]. The global distribution of tea plant is limited, as it is particularly sensitive to cold [10–12]. Cold injury has been shown to severely hinder the growth and yield of tea plant, with a notable example being a 56% reduction in average tea yield in China between 1990 and 2016 [13]. Consequently, enhancing the cold resistance of tea plant is paramount for minimizing yield losses and expanding suitable cultivation areas. To this end, various agronomic practices, such as irrigation, cover cropping, and the application of growth regulators, have been explored and found to offer some degree of protection against cold stress in tea plant [14–18]. However, comprehensive research on effective defensive measures remains limited, largely due to the complexity of the tea growing environment and the lengthy growth cycle of tea plant.

In recent decades, the focus has shifted toward plant resistance inducers, a novel class of pesticides that activate defense response mechanisms and bolster broad-spectrum resistance [19]. Natural inducers, classified based on their chemical properties, including oligosaccharides, chitosan, amino acids, proteins, polypeptides, nucleotide metabolites, glycoproteins, lipids, and glycopeptides [20, 21]. Increasing research has focused on the defensive capacity of plant resistance inducers in tea plant exposed to cold stress. For example, exogenous application of melatonin has been shown to improve the activity of antioxidant enzymes and maintain redox homeostasis in tea plant, thus mitigating cold-induced photosynthetic inhibition [22]. Similarly, chitosan oligosaccharide applications to tea plant under cold-stress stabilize chlorophyll levels, maintain photosynthesis, and increase antioxidative activities, such as CAT, POD, and SOD, as well as contents of other osmoregulatory substances like soluble sugars, proline, and soluble proteins [14]. Numerous studies have also demonstrated that amino acids can confer resistance to cold. γ-aminobutyric acid (GABA) effectively improves tea plant tolerance to cold stress by regulating various physiological and biochemical metabolic processes, including photosynthetic performance, membrane stability, and antioxidant activity [23]. The application of exogenous 5-aminolevulinic acid enhances cold tolerance in tea plant by increasing concentrations of phenolic compounds, particularly catechin, 3,4-dihydroxyphenylacetic acid, and procyanidin B2 [24]. Therefore, plant resistance inducers may have the potential to promote stress resistance in tea plant and can be harnessed in the development of new biopesticides with broad application prospects in tea cultivation.

An intriguing aspect of 2-amino-3-methylhexanoic acid (AMHA)-induced responses is that they confer protection against an extraordinarily wide range of biotic and abiotic stresses [25]. Our previous research has demonstrated the physio-biochemical responses of tea plant to AMHA under heat stress [10]. However, despite these promising findings, the fundamental genetic mechanisms underlying the relationship between AMHA and cold resistance in tea plant remain elusive. Given these findings, we hypothesize that AMHA may enhance cold stress resistance in tea plant, providing an appropriate, cost-effective, and practical approach to improving the crop's survival under cold conditions. To validate this hypothesis, we aim to comprehensively investigate the effects of AMHA application on the morphological, physiological traits, as well as the gene transcription profiles, of tea plant under cold stress conditions. The ultimate goal is to provide a cost-effective and practical solution for improving the survival and productivity of tea plant under cold stress.

## Results

### AMHA alleviates leaf injury in cold-stressed tea plant

To determine whether AMHA enhances cold stress resistance in tea plant, *C. sinensis* leaves were pretreated with various concentrations of AMHA prior to exposure to extreme low temperatures (Fig. S1a). Our results indicate that AMHA-pretreated plants at concentrations of 1, 10, and 100 nM, respectively, significantly enhanced their resistance against cold stress (−4°C) compared to mock-pretreated plants (0.02% Tween-20), as evidenced by changes in phenotype (Fig. S1a and b), OJIP curves (Fig. S1c), and PI_ABS_ (Fig. S1d). Notably, a concentration-dependent response was observed, with 100 nM AMHA identified as the optimal dosage for subsequent analyses.

**Figure 1:**
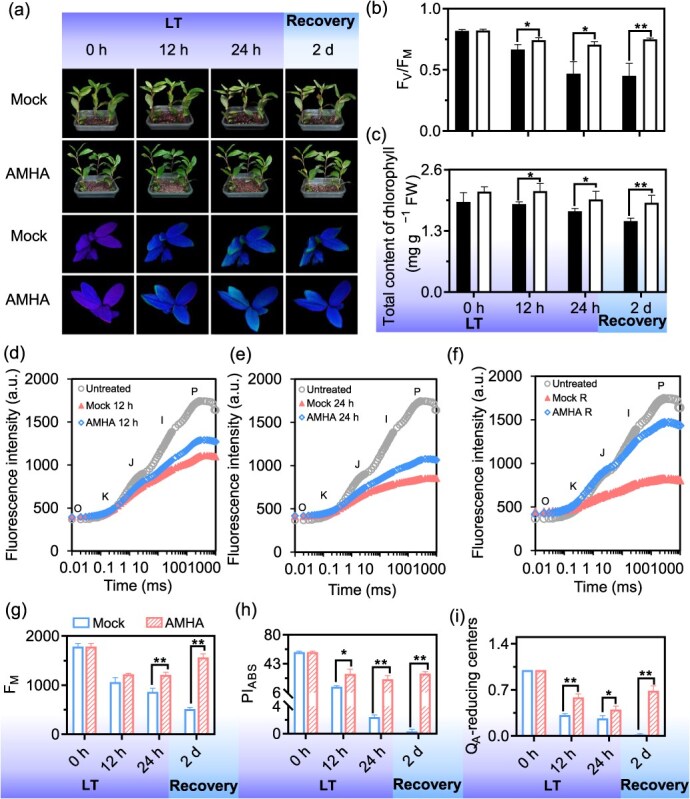
Effects of AMHA treatment on the phenotypes and photosynthetic performance of tea plant at different times under cold stress conditions. (a) Phenotypic changes and pseudo-color images of 1-year-old tea plant treated with AMHA under cold stress. Scale bars: 2 cm. (b) Statistical analysis of F_V_/F_M_ values in AMHA or mock-treated plants. The pseudo-color gradient indicates damage levels, with 1 indicating no damage and 0 indicating severe damage. (c) Chlorophyll content. (d–f) Raw fluorescence rise kinetics of AMHA-treated plants at 12 and 24 hours of post-cold stress and after 2 days of recovery. Untreated plants were healthy tea plants. (g) Maximal fluorescence at the peak P of OJIP (F_M_). (h) Performance index (PI_ABS_) representing overall photosynthetic efficiency. (i) The amount of PSII active RCs (Q_A_-reducing centers). Values indicate means ± SD (*n* = 3, ^*^P < 0.05, and ^**^P < 0.01, Student’s *t*-test), and this method was utilized for significance analyses below.

Under cold stress conditions, substantial phenotypic differences were noted between AMHA- and mock-pretreated plants. The top leaves of mock were severely frozen after 24 hours of cold treatment, with leaf margins turning brown and wilting until they either shriveled or fell off (Fig. 1a). In the case of AMHA pretreatment, the cold stress-induced damage was alleviated over time in tea plant. Leaf damage was further monitored by chlorophyll fluorescence imaging since photosynthetic activity is highly sensitive to cold stress. Following cold stress, the maximum quantum yield of PSII, F_V_/F_M_, in AMHA-treated plants showed a slight decrease, but remained significantly higher than that of the mock. Notably, the F_V_/F_M_ value of AMHA-treated plants increased by 50% relative to mock after 24 hours of cold stress and by 66% after 2 days of recovery (Fig. 1b). Additionally, chlorophyll content was consistently higher in AMHA-treated plants post-cold stress, with increases ranging from 12% to 29% compared to mock-treated plants (Fig. 1c). These results indicate that AMHA pretreatment effectively preserves the normal phenotype and photosynthetic activity of tea plant, significantly reducing severe leaf damage caused by cold stress.

Cold stress led to a distinct decrease in the fluorescence intensity of the OJIP curves, which was alleviated by AMHA (Fig. 1d–f). Specifically, the maximal fluorescence intensity, as indicated by the F_P_ value (F_M_), was increased by 16%, 26%, and 76% in AMHA-treated plants, compared to the mock at the indicated times after cold stress, respectively (Fig. 1g). This suggests that AMHA mitigates cold stress-induced damage to the photosynthetic activity of tea plant. Analysis using JIP-test parameters (Table S1) revealed that the PSII performance index (PI_ABS_) was highly sensitive to stress intensities [26]. The fraction of Q_A_-reducing centers was dramatically decreased after cold stress in tea plant, but was mitigated by AMHA. During recovery, a normal level of the values of these two parameters was almost regained in AMHA-treated plants, whereas the recovery of mock-treated plants was insignificant (Fig. 1h and i). The number of Q_A_-reducing centers increased by 85%, 49%, and 616% in AMHA-treated plants compared to mock at 12 h, 24 h after cold stress, and 2 d of recovery, respectively. The performance index of the active oxygen evolution complex (OEC) centers in AMHA-treated plants was increased by 30%, 74%, and 312% compared to mock at the indicated time, respectively (Fig. S2a). Further, the PI_ABS_ values of the AMHA-treated plants were 121% and 86% greater than of mock at the indicated time after cold stress, respectively, while mock-treated plants exhibited nearly zero PI_ABS_ values after 2 days of recovery. This indicated that AMHA alleviated the diminution in the overall activity of PSII activity caused by cold stress in tea plant.

The ABS/CS and TR_0_/CS of AMHA-treated plants were significantly higher than those of mock-treated plants after cold-stress exposure, indicated enhanced light absorption, energy capture, and electron transfer efficiency (Fig. S2b and c). ET_0_/CS, φ_Eo_, and ψ_Eo_ also increased in AMHA-treated plants compared to mock (Fig. S2d–f). Notably, ET_0_/CS were 127% higher in AMHA-treated plants post-cold stress. The heat dissipation energy and the number of PSII reaction centers in tea leaves significantly increased in the AMHA-treated plants, boosting electron transport chain activity. The RE_0_/RC, φ_Ro_, and PI_total_ values of AMHA-treated plants were significantly higher by 169%, 159%, and 240% compared with the mock at the indicated time after cold stress, respectively (Fig. S2g–i). These results indicate that AMHA promotes photosynthetic capacity, ultimately enhancing the photosynthetic efficiency of tea leaves.

### AMHA enhances osmotic adjustment, mitigates excessive ROS accumulation and boosts antioxidant defense in cold-stressed tea plant

**Figure 2:**
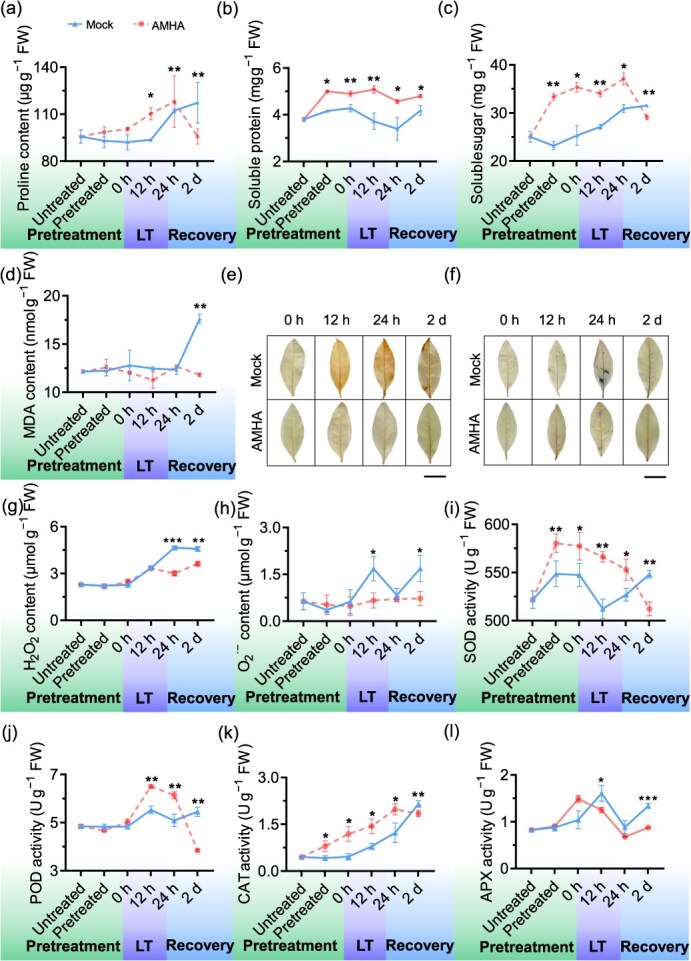
Dynamic effects of AMHA on osmotic adjustment substances contents, ROS levels, and antioxidant accumulation in tea plant during pretreatment, low temperature (LT), and recovery stages. (a–d) Contents of proline (a), soluble sugar (b), soluble protein (c), and MDA (d). (e) and (f) Accumulation of H_2_O_2_ and O_2_^**·−**^ detected by NBT and DAB staining, respectively. Scale bars: 2 cm. (g) and (h) Contents of H_2_O_2_ and O_2_^**·−**^. (i-l) Enzyme activities of SOD (i), POD (j), CAT (k), APX (l). Values indicate means ± SD (*n* = 3, ^*^P < 0.05, ^**^P < 0.01, and ^***^P < 0.001, Student’s *t*-test)

Lipid peroxidation, a key indicator of cold injuries, can be quantified by malondialdehyde (MDA) levels. Osmotic adjustment substances such as proline, soluble protein, and soluble sugar were examined [2]. We observed that the contents of proline, soluble protein, and soluble sugar significantly increased in AMHA-treated plants post-cold stress exposure compared to mock-treated plants (Fig. 2a–c). Specifically, after 12 hours of cold stress, AMHA-treated plants showed significant increase of 18%, 36%, and 26% in proline, soluble protein, and soluble sugars contents, respectively, compared to mock. Interestingly, MDA content remained near-normal levels in AMHA-treated plants (Fig. 2d), whereas a remarkable accumulation was observed in mock-treated plants. The reduced MDA content in AMHA-treated plants during recovery likely contributed to maintaining normal osmotic potential, thereby mitigating cold stress-induced leaf damage. These findings suggest that AMHA pretreating enhances osmotic regulation capacity, reduces lipid membrane peroxidation, and consequently bolsters cold resistance in tea plant.

Following cold stress exposure, brown and dark blue deposits were observed in mock-treated tea plants after DAB and NBT staining, respectively (Fig. 2e and f), indicating higher ROS accumulation in mock-treated tea plant compared with the AMHA-treated tea plant. H_2_O_2_ and O_2_^**·−**^ changes of AMHA-treated plants were 35% and 60% lower, respectively, compared to mock at the indicated time post-cold stress (Fig. 2g and h).

Antioxidative enzymes play a pivotal role in reducing ROS accumulation induced by cold stress [27]. POD activity in AMHA-treated plants had little change, while CAT and SOD activity were higher in AMHA-treated plants than in mock-treated plants post-cold stress (Fig. 2i–l). The activities of POD, SOD, CAT, and APX in AMHA-treated plants showed higher activities than in mock at the indicated time points. Additionally, the expression levels of the four antioxidant enzyme-related genes *CsSOD*, *CsAPX*, *CsGST*, and *CsGR* in AMHA-treated plants were significantly higher compared to mock at the indicated time (Fig. S3). These results further indicate that AMHA enhances cold resistance in tea plant by boosting their ROS scavenging capacity.

**Figure 3:**
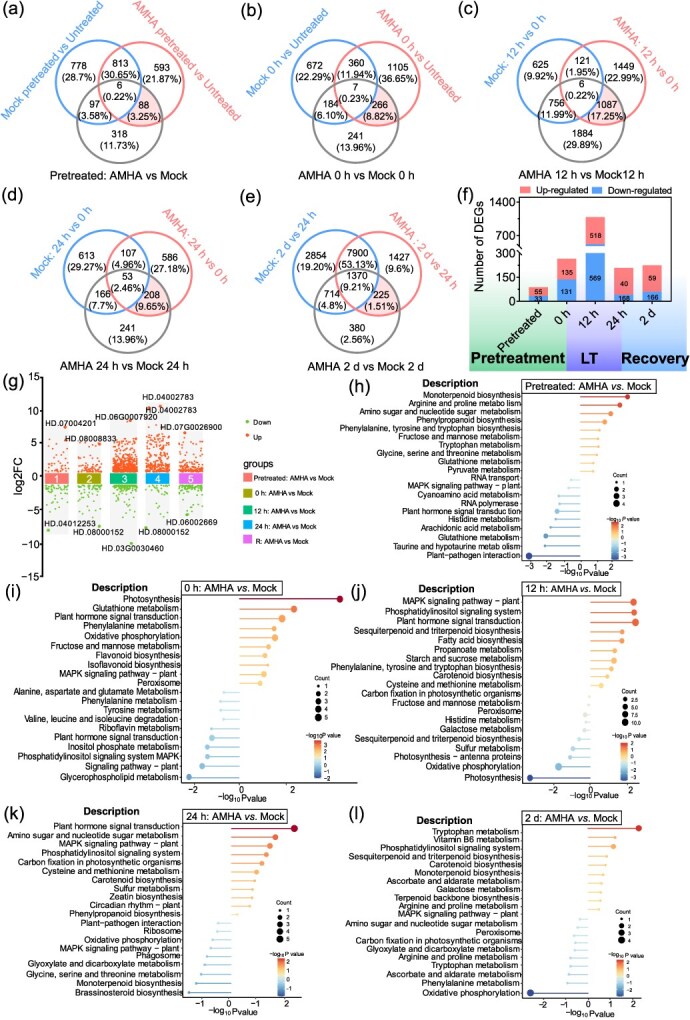
Identification and functional enrichment analysis of DEGs in AMHA-treated plants compared to mock-treated plants. (a–e) Venn diagrams showing common and unique DEGs in AMHA- or mock-treated plants during the pretreatment, cold stress, and 2 days of recovery stages. (f) Bar plot indicating the total number of DEGs identified in the comparisons outlined. (g) Volcano plots displaying DEGs. (h, i) Lollipop plots displaying KEGG enrichment scores based on enrichment score (ES) ranking derived from differential expression analysis.

### Transcriptomic analysis of cold resistance mechanisms induced by AMHA in tea plant

Principal component analysis of gene expression profiles from 33 samples yielded 11 components (Table S2, Fig. S4). In comparison to mock-treated plants, 88 and 266 differentially expressed genes (DEGs) were detected in the AMHA-treated plants at the indicated time of the pretreatment stage (Fig. 3). A total of 1087, 208, and 225 DEGs were identified in the AMHA-treated plants at the indicated time of cold stress and at 2 days of recovery, respectively.

AMHA-induced genes were enriched in gene ontology (GO) terms associated with defense-related metabolic pathways at the indicated time of AMHA pretreatment stage. These include glutathione metabolic process, AsA biosynthetic process, arginine catabolic process to glutamate, phospholipid metabolic process, and lipid metabolic process (Tables S3–S6, Fig. S5a and b). Notably, upregulated AMHA-induced genes during the pretreatment were primarily involved in processes related to the L-AsA biosynthetic process. In response to cold stress, AMHA-induced genes in the tea plant were specifically enriched in biological process terms related to photosynthesis, defense response, response to cold, cold acclimation, glucosinolate metabolic process, response to water deprivation, and hormone-mediated signaling pathway (Fig. S5c and d). Surprisingly, compared to 24 hours of cold stress, AMHA-induced genes were involved in responses of the photosynthetic system, such as photosynthetic electron transport chain, photosynthetic electron transport in PSII, and light reaction at 2 days of recovery (Fig. S5e). Overall, the GO enrichment results indicate that AMHA promoted defense mechanisms in tea plant against cold stress exposure.

Kyoto Encyclopedia of Genes and Genomes (KEGG) pathways analysis revealed that up-regulated DEGs in tea plant during AMHA pretreatment were primarily included glutathione metabolism, phenylpropanoid biosynthesis, and ascorbate and aldarate metabolism (Fig. 3h and i, Tables S5 and S6). The DEG pathways of the tea plant under cold stress were enriched in flavonoid and carotenoid biosynthesis (Fig. 3j and k). At 2 days of recovery, the highly represented pathways of tea plant DEGs were tryptophan metabolism, vitamin B6 metabolism, and phosphatidylinositol signaling system (Fig. 3l). Among down-regulated DEGs, metabolite and stress-related KEGG terms were enriched, with the plant hormone and MAPK signaling were identified as major regulatory pathways during AMHA pretreatment. KEGG term enrichment for cold stress mainly included defense-related KEGG terms, such as, oxidative phosphorylation, and photosynthesis. Additionally, ROS scavenging, carotenoid biosynthesis, and ascorbate and aldarate metabolism showed enrichment after cold stress and at 2 days of recovery. Generally, AMHA-treated plants exhibited more significant alterations in DEGs compared to mock. The enriched pathways were primarily related to the photosynthetic system and secondary metabolic pathways.

**Figure 4:**
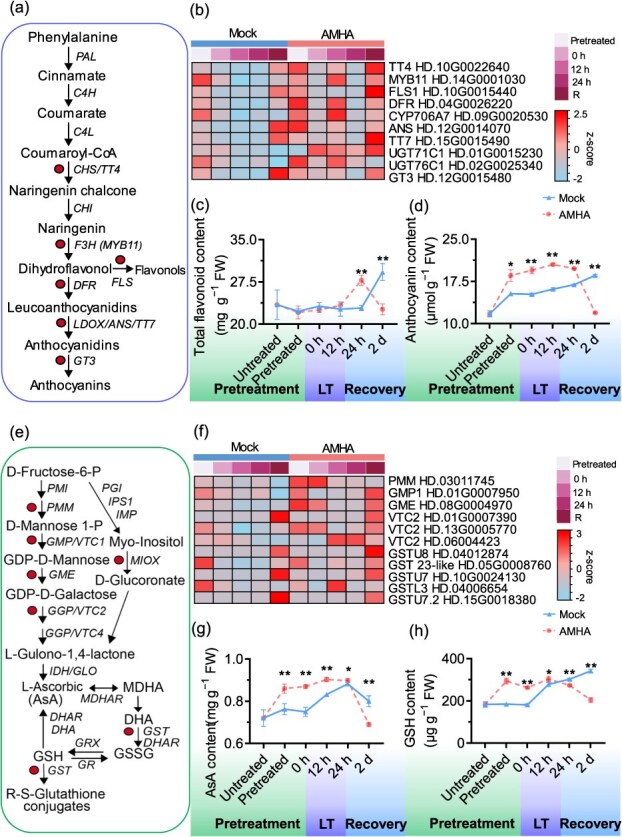
Effects of AMHA on flavonoid/anthocyanin and AsA metabolic pathways in tea plant. (a) and (b) Metabolism pathway and transcriptional levels of DEGs in the flavonoid/anthocyanin. (c) and (d) Total contents of flavonoid and anthocyanin, respectively. (e) and (f) metabolism pathway and transcriptional levels of DEGs in the AsA metabolism pathway. (g) Total contents of GSH and AsA, respectively. Values indicate means ± SD (*n* = 3, ^*^P < 0.05, and ^**^P < 0.01, Student’s *t*-test), and this method was utilized for significance analyses below.

The anthocyanin metabolic pathway was induced in AMHA-treated plants in response to cold-stress exposure. The pathway encompassed 10 DEGs, including TRANSPARENT TESTA 4 (*TT4*), flavonol synthase (*FLS*), flavanone 3-hydroxylase (*DFR*), TRANSPARENT TESTA (*TT7*), *CYP706A7*, anthocyanin synthase (*ANS*), *UGT71C1*, *UGT76C1*, *MYB11*, and *GT3* (Fig. 4a and b). Numerous anthocyanin pathway-related genes were up-regulated during cold stress. Notably, AMHA-treated plants accumulated AsA during cold-stress, acting as a scavenger to protect cells from stress and damage by eliminating free radicals [6]. The expression profiles of AsA biosynthetic genes showed that six genes, including three Vitamin C Defective 2 (*VTC2*), GDP-Mannose-3′,5′-Epimerase (*GME)*, Mannose-1-Phosphate Mutase (*PMM)*, and GDP-Mannose Pyrophosphorylase (*GMP*), were associated with AsA synthesis (Fig. 4e and f). Prior to cold stress exposure, most carotenoid biosynthesis genes exhibited slight change in the expression levels. However, during cold stress exposure, these genes were significantly up-regulated in tea plant. Specifically, *β*-carotene levels were significantly higher in AMHA-treated plants compared to mock after exposure to cold stress (Fig. S6). These findings suggest that AMHA’s effects on secondary metabolism biosynthesis may substantially enhance the transcriptional levels of the genes linked to cold resistance.

### Time-ordered co-expression network analysis of cold-responsive genes in AMHA-treated tea plant

**Figure 5:**
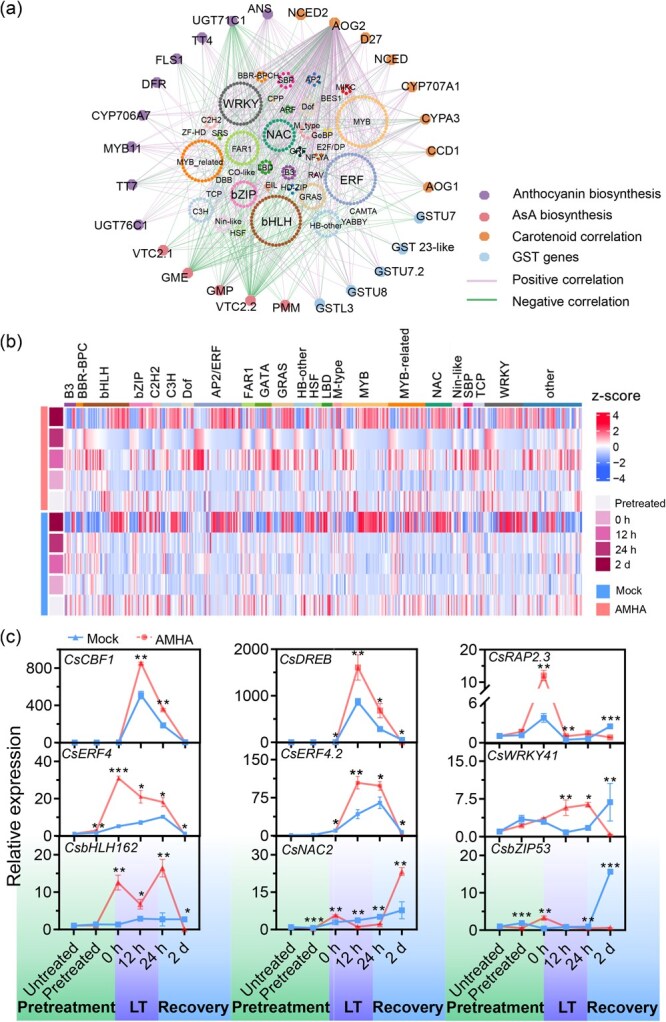
Analysis of transcription factors (TFs) potentially correlated with flavonoid/anthocyanin, carotenoid, and AsA biosynthesis, as well as *GST* expression in AMHA-treated plants. (a) Diagram depicting the transcriptional regulatory network of flavonoid/anthocyanin, carotenoid, and AsA biosynthesis pathways, as well as *GSTs*. Non-TFs are represented by hexagons; TFs are depicted by different circles, with interconnecting lines showing potential co-expression associations between TFs and non-TF genes. (b) Heatmap displaying differentially expressed TFs following AMHA exposure. (c) RT-qPCR validation results of key TFs. Values indicate means ± SD (*n* = 3, ^*^P < 0.05, ^**^P < 0.01, and ^***^P < 0.001, Student’s *t*-test)

Defense-related metabolic substances, such as flavonoids/anthocyanins, carotenoids, and AsA, play significant roles in resisting oxidative damage caused by cold stress. This prompted us to determine whether flavonoid/anthocyanin-, carotenoid-, and AsA pathway-related genes exhibited temporal expression patterns. A regulatory network encompassing transcription factors (TFs) and DEGs in the flavonoid/anthocyanin, carotenoid, and AsA pathways, as well as glutathione S-transferase (*GST*) genes, was constructed (Fig. 5a). A total of 494 TFs were identified, which were primarily regulated and the closely connected to TFs, including *CsbHLHs*, *CsbZIPs*, *CsDofs*, *CsERFs*, *CsMYBs*, *CsNACs*, and *CsWRKYs* (Fig. 5b). Among them, the transcript abundances of the TFs *CsCBF1* (HD.06G0016360), *CsDREB* (HD.01G0015340), *CsRAP2.3* (HD.10G0029300), *CsERF4* (HD.10G0029300), *CsERF4.2* (HD.02G0007290), *CsWRKY41* (HD.12G0016750), *CsbHLH162* (HD.04G0018140), *CsNAC2* (HD.09G0009680), and *CsbZIP53* (HD.08G0000880) were significantly different in AMHA- or mock-treated tea plant, exhibiting at least three-fold higher expression under cold stress conditions. Specifically, the expression levels of *CsCBF1*, *CsDREB*, *CsRAP2.3*, *CsERF4*, *CsERF4.2*, and *CsWRKY41* were significantly upregulated in AMHA-treated plants after cold stress compared to mock, whereas *CsbHLH162* and *CsNAC2* were significantly downregulated. These TFs were validated using RT-qPCR (Fig. 5c). Further evidence supporting this regulatory mechanism was provided by the *cis*-elements in the promoter region (Fig. S7). The complex regulatory network analysis showed that the *ERF* TF family was positively correlated with the expressed profiles of DEGs in the flavonoid/anthocyanin, carotenoid, and AsA pathway, as well as with *CsGSTs* expression. These results indicate that *ERFs* positively regulate related secondary metabolic pathways and *CsGST* expression.

### AMHA modulates ROS levels and antioxidant accumulation through *CsGSTs*

**Figure 6:**
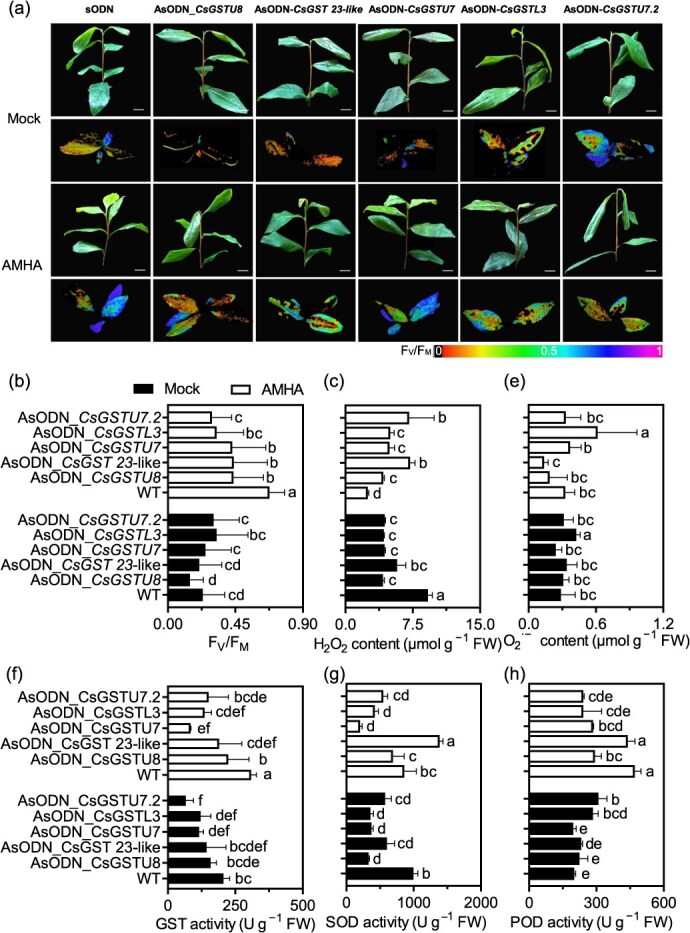
Effects of AMHA on ROS homeostasis in tea plant of AsODN_GSTs under cold stress conditions. (a) Phenotype and photosynthetic pigments of sODN and AsODN_*GSTs* of AMHA or mock-treated plants. Scale bars: 1 cm. (b) F_V_/F_M_ value differences between sODN and AsODN_*GSTs* of AMHA or mock-treated plants. (c) and (d) H_2_O_2_ and O_2_^**·−**^ contents of sODN and AsODN_*GST* of AMHA or mock-treated plants. (e-g) Changes in GST, SOD, and POD activities in sODN and AsODN_*GSTs* of AMHA or mock-treated plants. Data are presented as means ± SEs (15 biological replicates). Different lowercase letters indicate statistically significant differences by the LSD multiple range test (*P* < 0.05).

To confirm the role of *GST* genes in response to AMHA during cold stress, the expression patterns of *CsGSTs* were analyzed. Results revealed that *CsGSTU8*, *CsGST 23-like*, *CsGSTU7*, and *CsGSTL3* exhibited higher expression levels in AMHA-treated plants compared to mock after 12 hours of cold stress (Fig. S8a–e). Furthermore, *CsGSTU8*, *CsGST 23-like*, *CsGSTU7*, and *CsGSTU7.2* were upregulated in AMHA-treated plants under cold-stress conditions. The expression levels of AsODN*_CsGSTU8*, AsODN*_CsGST 23-like*, AsODN*_CsGSTU7*, AsODN*_CsGSTL3*, and AsODN*_CsGSTU7.2* were significantly lower compared to sODN (Fig. S8f). Phenotypic differences in *CsGSTU8*, *CsGST 23-like*, *CsGSTU7*, *CsGSTL3*, and *CsGSTU7.2* were examined in sODN- and AsODN_GST-plants, both with and without AMHA treatment (Fig. 6a and b). After 12 hours of cold stress, all AsODN_GST tea plant showed cold-hypersensitive phenotypes, characterized by increased wilting of young shoots and lower F_V_/F_M_ values compared to sODN, particularly those receiving AMHA treatment (Fig. 8a and b). In AMHA-treated plants of sODN, the levels of H_2_O_2_ and O_2_^**·−**^ were lower than those of the AsODN in mock-treated plants, whereas the activities of GST, SOD, POD, and CAT were increased (Fig. 6c–h, Fig. S8g). Remarkable damage was observed in mock-treated AsODN_CsGSTU7 plants. Collectively, our findings indicate that suppressing *CsGSTs*, specifically *CsGSTU7*, enhances enzymatic activities and ROS scavenging capacity in tea plant, thereby improving their resilience to cold stress.

### AMHA promotes ROS detoxification via *CsGSTU7* in cold-stressed tea plant

**Figure 7:**
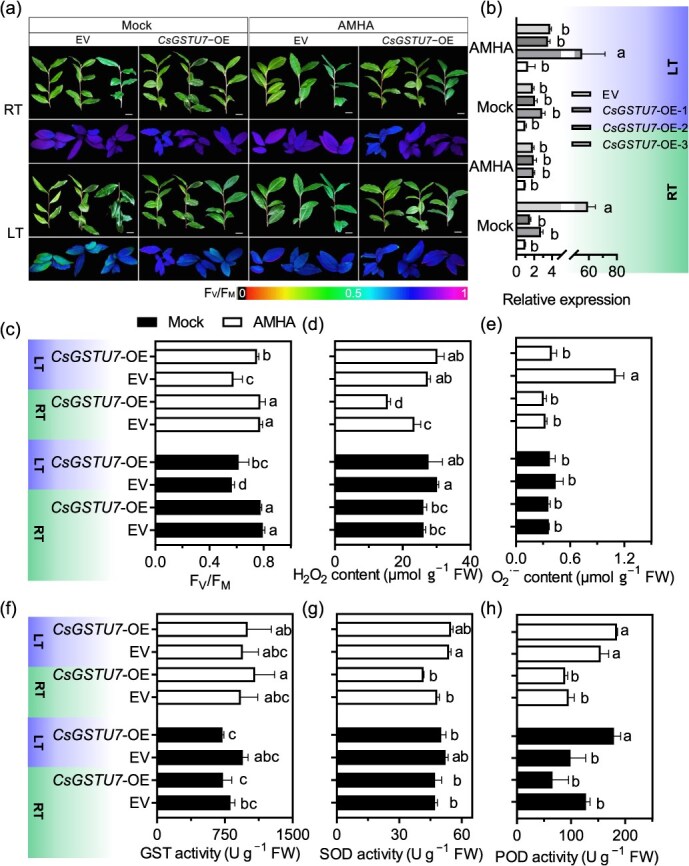
Overexpression of *CsGSTU7* promoted stress resistance in transgenic tea plant under cold stress. (a) Phenotypes under room- and low-temperature conditions with AMHA or mock-treated plants. Scale bars: 1 cm. (b) Expression pattern analysis of the *CsGSTU7* under room- and low-temperature conditions. (c-d) Measurement of H_2_O_2_ and O_2_^**·−**^ contents under room- and low-temperature conditions. (f-h) Measurement of GST, SOD, and POD activities under room- and low-temperature conditions. The values are presented as the means ± SDs of three biological experiments. Different lowercase letters indicate statistically significant differences by the LSD multiple range test (*P* < 0.05).

To further explore the role of *CsGSTs* expression in the resistance of tea plant to cold stress, we incubated *CsGSTU7*-overexpressing (OE) lines and empty vector (EV)-transformed lines under cold stress (−4°C) for 12 hours. After cold exposure, EV plants exhibited wilted and shrunken leaf phenotypes (Fig. 7a and b). Notably, AMHA pretreatment enhanced cold resistance in tea plant overexpressing *CsGSTU7*. Moreover, the content of H_2_O_2_ and O_2_^**·−**^ was significantly higher in *CsGSTU7*-OE plants compared to EV plants (Fig. 7d and e). Under room-temperature (RT) conditions, slight differences were observed in the indices between the EV and *CsGSTU7*-OE plants. However, cold stress dramatically increased the activities of antioxidant enzymes such as GST, CAT, POD, and SOD, and these activities were higher in *CsGSTU7*-OE plants compared to EV plants (Fig. 7f–h, Fig. S9). These results confirm the involvement of *CsGSTU7* in regulating the enzymatic antioxidant defense system under cold stress.

## Discussion

Tea plant, known for their robust flavor and health-promoting properties, are predominantly distributed in tropical and subtropical areas. However, their productivity is severely constrained by environmental factors, particularly cold stress, which can cause substantial damage or even death to the plants [28, 29]. Consequently, there is a pressing need for further research to elucidate the underlying mechanisms involved in enhancing cold resistance in tea plant through the use of plant resistance inducers. Such inducers have been shown to positively regulate cold resistance in other plants, such as *Capsicum annuum* [30] and *Vitis vinifera* [31]. AMHA, a novel natural product, holds promise as a potential bio-stimulant to improve the cold stress resistance of tea plant. However, systematic investigations of the physiological and molecular mechanisms underlying the effects of AMHA on tea plant exposed to cold stress have been lacking. In this study, we explored the influence of AMHA pretreatments on the physiological responses and transcriptional regulation of tea plant under cold stress condition. The findings indicate that AMHA alleviates the adverse effects of cold stress and serves as an effective bio-stimulant for enhancing the performance of tea plant under cold-stress conditions.

### AMHA enhances cold resistance by maintaining higher photosynthetic activity

Photosynthesis, being highly sensitive to cold stress, serves as a crucial indicator of plant response [10, 32]. The rapid chlorophyll fluorescence technique was used to investigate the impact of cold stress on the photosynthetic activity of tea plant. The OJIP curve of the tea plant exhibited severely distortion in response to cold-stress exposure (Fig. 1d–f), transitioning from displaying multiple OJIP transients to converging into a smooth straight line, with a notable reduction in the P-point fluorescence value F_M_ [33]. However, AMHA pretreatment mitigated cold damage, as evidenced by the reduced deformation of the OJIP curve and higher P-point fluorescence values (Fig. 1g). This suggests that AMHA alleviates the detrimental effects of cold stress on photosynthetic machinery of tea plant.

Analyses of maximum photochemical efficiency and JIP-test parameters showed a significantly decreased in PI_ABS_ values during cold stress (Fig. 1h), indicating severe damage to PSII activity, which consequently led to a decrease in the photosynthetic rate [34]. In AMHA-treated plants, RE_0_/RC, representing the quantum yield of electron transfer per unit reaction center from Q_A_ to PSI electron acceptor, was significantly decreased under cold-stress conditions, indicating the inactivation of some PSII reaction centers in the leaves (Fig. S2h) [35]. This finding is consistent with the observations of Ashrostaghi *et al.* in cucumber and turfgrass [36]. Furthermore, the ABS/CS and TR_0_/CS in AMHA-treated plants significantly increased under cold-stress conditions, indicating that AMHA significantly enhances light absorption, energy capture, and electron transfer in tea plant (Fig. S2b and c). This moderation mitigated the severe damage caused by cold stress on the PSII receptor side, thereby improving the stability of the photosystem structure and electron transport chain. Thus, AMHA elevated heat dissipation energy and energy flux in PSII reaction centers, leading to an increased the number of reaction centers and further enhancing the photosynthetic efficiency of PSII in tea leaves. Furthermore, increases in RE_0_/RC, φ_Ro_, and PI_total_ in AMHA-treated plants indicate that AMHA promoted electron transfer to PSI, improves its electron transfer efficiency, and ultimately, enhances the photosynthetic efficiency of the tea leaves (Fig. S2h–j). This may be attributed to an increase in the number of PSI receptors due to higher electron acceptance on the PSI receptor side.

### AMHA-induced cold stress resistance in tea plant by enhancing osmotic adjustment ability

Metabolic reprogramming in response to cold stress is crucial for plant survival [37]. Our analysis of DEGs related to AsA metabolism, flavonoids/anthocyanins and carotenoids under cold stress revealed that AMHA positively modulated these pathways. AsA, a nonenzymatic antioxidant, plays a vital role in scavenging ROS and maintaining plant functions [38]. In pepper fruit, the treatment with glycine betaine has been shown to increase the activity of APX, GR, MDHAR, and DHAR, along with the expression of their encoding genes. This facilitates the regeneration of two non-enzymatic antioxidants, AsA and GSH, effectively eliminates accumulated H_2_O_2_, and thereby enhances cold resistance [39]. Recent reports have emphasized the role of AsA in enhancing salt tolerance in tomatoes through the *SlZF3* gene, which regulates AsA synthesis [40], and in mediating cold stress via the AcePosF21-AceGGP3 module in kiwifruit [41]. Our results indicated that AMHA upregulates genes involved in the AsA-GSH pathway, including *CsVTC2*, *CsGME*, *CsPMM*, and *CsGMP*, indicating a crucial role for AMHA in modulating these pathways in treated plants.

Flavonoids/anthocyanins, as important secondary metabolites in tea plant, not only contribute to tea characteristics but also reflect the vegetative growth of plants [42, 43]. After cold stress, the contents of flavonoid and anthocyanin were significantly higher in AMHA-treated plants compared to mock (Fig. 4a). Exogenous abscisic acid alleviates cold damage in bananas by regulating anthocyanin accumulation through the activation of *MaABI5*-like expression [44]. Furthermore, GABA treatments elevate flavonoid content and modify related gene profiles in tea plant during cold stress exposure [45]. The crucial roles of *CsPAL*, *Cs4CL*, and *CsCHS* in flavonoid biosynthesis in tea plant under cold stress have been well reported [46]. Here, we identified genes encoding key enzymatic components in tea plant during cold stress and found 10 DEGs enriched in flavonoid biosynthesis in AMHA-treated plants (Fig. 4). TFs may significantly impact the anthocyanin accumulation. In tomato, *SlAREB* was shown to regulate *SlDFR* and *SlF3′5′H* and improve anthocyanin accumulation under cold stress [7].

One of the most significant effects of AMHA-induced signaling is the promotion of carotenoid biosynthesis. Carotenoids provide considerable photoprotection and possess antioxidant capacity, primarily functioning within the leaf plastids [47, 48]. In tea plant, *CsCBF2* negatively regulates the transcriptional activity of the carotenoid pathway gene *CsZEP1*, which plays a crucial role in the tea plant’s response to cold stress [49]. Our results indicate that carotenoid biosynthesis plays a crucial role in enhancing cold resistance in *C. sinensis* (Fig. S6). In line with the antioxidant function of carotenoids, the GO pathway ‘response to oxygen-containing compound’ is enriched in AMHA-treated plants (Table S4, Fig. S5). Co-expression analysis results and quantitative content analysis in AMHA-treated plants further support our hypothesis that the antioxidant capacity induced by AMHA protects tea plant during cold stress exposure. AMHA pretreatment of tea plant increased AsA, flavonoid/anthocyanin, and carotenoid production, which could affect the plant's biological processes and resistance to cold stress.

### AMHA improves redox balance to enhance cold stress resistance

Plants produce a variety of antioxidant components to defend against changing ambient conditions [50]. Cold stress triggers an imbalance in ROS, affecting tea plant growth, enzyme activities, photosynthetic activity, and osmotic regulatory capabilities [46]. However, AMHA significantly attenuates injury-related phenotypes and chlorophyll fluorescence (Fig. 1), indicating its role as a plant inducer associated with various stresses in plants [51]. AMHA is associated with increased activities of antioxidative systems, including CAT, POD, and SOD (Fig. 2i–k). Higher antioxidant enzyme activities indicate the effective elimination of free radicals produced by cold-stress conditions [52].

Additionally, the AMHA-induced ROS response is activated. ROS bursts are caused by damage to photosynthesis in the chloroplasts [53, 54]. AMHA can exert its effects beyond the chloroplast, mitigating the excessive production of ROS. Consequently, this triggers a cascade of stress-related ROS responses, encompassing the increase in GST activity as well as the accumulation of GSH and AsA. As shown in Fig. 2, AMHA ameliorates oxidative stress under cold-stress conditions by restraining high activities of antioxidants and increasing the contents of GSH and ascorbate metabolism in ROS-eliminating pathways. A significant proportion of the accumulations of GSH and AsA serves to delay aging, regulate plant growth, and scavenge ROS produced by stress. In our research, ROS generation was evidenced by high accumulations of H_2_O_2_ and O_2_^**·−**^, which likely induced the elevated GSH and AsA contents in tea plant.

### GST plays a critical role in AMHA-induced cold resistance in tea plant

Plant GSTs are glutathione-dependent enzymes involved in peroxide detoxification [55]. Some reports have indicated that the expression of *GST* genes is upregulated in response to cold stress in various plants, such as *Cucumis sativus* [33], *Saccharina japonica* [56], and *Poncirus trifoliata* [15]. sODN_CsGST plants accumulated significantly lower levels of ROS, while antioxidant activities of SOD and POD were noticeably increased. GSTU, a plant-specific GST, is known to play a pivotal role in the scavenging of ROS [57]. Previous studies have indicated that cold stress strongly induces the expression of *CsGSTU47* and *CsUGTL6* in tea plant [58], implying that GSTs from tea plant may have conserved functions in response to cold stress. *CsGSTU7* is rapidly induced under diverse environmental conditions and is responsive to salicylic acid and other phytohormones [59]. In this study, we observed that *CsGSTU7*, specifically *CsGSTU7* (HD.10G0024130) and *CsGSTU7.2* (HD.15G0018380), were strongly induced by AMHA under cold stress. Several GSTs have been implicated in ROS scavenging and stress response mechanisms [15, 33, 56]. GST proteins catalyze the conjugation of GSH to various hydrophobic and electrophilic substrates, including ROS, thereby protecting the cell from oxidative burst. During catalysis, the conserved GSH binding site (G-site) governs the binding and correct orientation of GSH, while the substrate binding pocket (H-site) aids in substrate binding by providing a hydrophobic environment [60, 61]. Our results suggest that *CsGST* genes, including *CsGSTU8*, *CsGST 23-like*, *CsGSTU7*, *CsGSTL3*, and *CsGSTU7.2*, which possess the conserved GSH binding site and substrate binding pocket, have the potential to catalyze the conjugation of GSH to diverse substrates. This potential was further validated by the higher GST activity observed in transgenic plants compared to WT plants, which was accompanied by enhanced ROS degradation in response to cold stress (Fig. 6c and e).

Research has shown that GSTs are subject to direct regulation by various transcription factors, including ERF, bHLH, and NAC types, in plants under cold stress conditions [15, 57, 62]. In *Poncirus trifoliata, PtrERF9*-activated *PtrGSTU17* expression was enhanced through the regulation of ROS degradation to cope with cold stress [15]. In line with our prediction results (Fig. S7), certain TFs that regulate stress and defense responses have been verified. Information on upstream TFs of *CsGST* is available, and we predict that TFs such as ERF, WRKY, and Dof may serve as upstream regulators of *CsGSTs*. While the specific regulatory relationship between these TFs and *CsGSTs* has not been confirmed, our results provide a direction for future investigation. Overexpression of *CsGSTU7* in transgenic tea plant conferred enhanced cold resistance, evidenced by reduced ROS accumulation and increased antioxidant enzyme activities (Fig. 7f–h)*.* This suggests that the central role of GSTs in AMHA-induced cold resistance, functioning synergistically with the AsA-GSH cycle to maintain redox balance.

**Figure 8:**
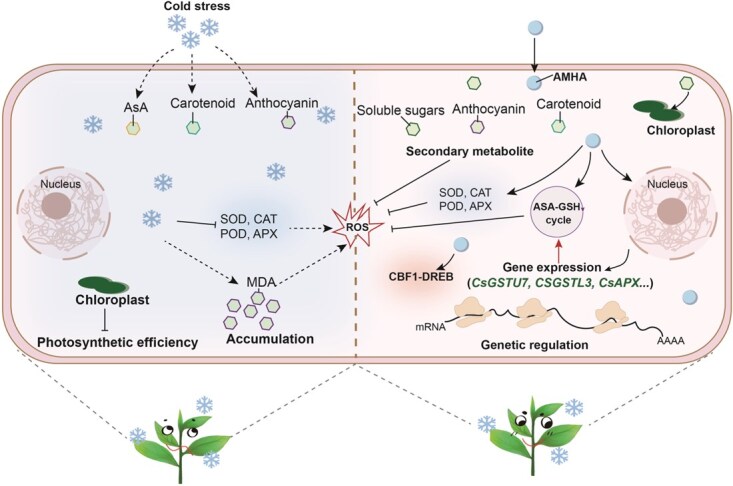
A putative regulatory work model showing how AMHA is involved in mitigating cold damage in tea plant. AMHA pretreatment elicits physiological responses, including enhanced antioxidant enzymes, chloroplast protection, and osmotic adjustment, while also affecting the expression levels of glutathione S-transferase (*GST*). AMHA significantly increases the levels of catalase (CAT), peroxidase (POD), superoxide dismutase (SOD), ascorbate peroxidase (APX), anthocyanins, β-carotenes, ascorbic acid (AsA), glutathione (GSH), and soluble sugars, which are related to cold stress resistance. This alleviates physiological and metabolic damages, such as impaired photosynthetic efficiency, increased reactive oxygen species (ROS), and disrupted osmotic stability, caused by cold stress. AMHA mitigates the damage of cold stress by inducing the expression levels of *CsGSTU7* and other key genes, as well as by stimulating the synthesis of the AsA-GSH system. AMHA maintains normal tea plant growth under cold-stress exposure by inducing genes related to cold resistance and osmolyte production.

In summary, our study demonstrates that pretreatment of tea plant with AMHA effectively enhances their physiological resilience to cold stress-induced damage. AMHA upregulates the expression of *GST* genes, particularly *CsGSTU7*, which plays a key role in promoting synergistic interactions with the AsA–GSH cycle and elevating antioxidant capacity. This mitigates lipid membrane peroxidation damage, maintains normal chlorophyll synthesis and photosynthesis, strengthens cold resistance, and promotes tea plant growth. Furthermore, AMHA regulates the biosynthetic pathways of key secondary metabolites, such as flavonoids/anthocyanins, carotenoids, and soluble sugars, further enhancing cold stress resistance (Fig. 8). Our study provides profound mechanistic insights into the utilization of AMHA pretreatment to mitigate cold stress in tea plant.

## Materials and methods

### Plant materials

Healthy, one-year-old *C. sinensis* ‘Baiye 1’ cuttings were maintained under room temperature (RT) conditions at the Nanjing Agricultural University research station, located at GPS coordinates 32.2625°N, 118.502347°E. The soil substrates for tea plant growth comprised a blend of soil, vermiculite, and perlite in a 3:1:1 ratio. To determine the optimal concentration of AMHA for enhancing cold resistance, tea plant was pretreated with various concentrations of AMHA (1, 10, and 100 nM) using a fine mist sprayer, with 0.01% Tween-20 serving as a surfactant. The mock treatment involved the application of an equivalent volume of 0.01% Tween-20 alone.

### Experimental treatment

The ‘Baiye 1’ tea cuttings were sprayed with 100 nM AMHA twice before cold-stress treatments, with a 24-hour interval between treatments. Tea plant treated with an equivalent concentration of 0.01% Tween-20 served as the mock. Leaf samples were collected at 12 and 24 hours after AMHA pretreatment. The remaining cuttings, from which leaves were not collected, were exposed to continued cold stress conditions for an additional 12 and 24 hours, respectively. For the cold stress treatment, all tea plant were maintained in a −4°C growth chamber for 24 hours under white light (150 μmol∙m^−2^∙s^−1^) with a 10:14-hour photoperiod. Recovery samples were harvested after 2 days in RT condition, following the completion of the cold stress treatment. The experimental design is depicted in Fig. S10. At each sampling point, three biological replicates were collected for the purpose of physiological, molecular, and target metabolite analyses. These samples were immediately stored at −80°C with liquid nitrogen for subsequent experiments.

### Measure of chlorophyll (Chl) *a* fluorescence rise kinetics OJIP

OJIP curves, representing the Chl *a* fluorescence rise in fully expanded mature tea leaves, were analyzed using a Plant Efficiency Analyzer (Handy-PEA fluorometer, Hansatech Instruments Ltd., King’s Lynn, Norfolk, UK). The analysis was conducted with 1 s pulses of red light at 650 nm, with an intensity of 3500 μmol (photons) m^−2^∙s^−1^. The tea plants were dark-adapted for 30 minutes at 25°C before measurement. Subsequently, the chlorophyll fluorescence signal was recorded for 1 second. A total of 15 biological replicates were used for the tests. Detailed equations, formulations, and definitions of JIP-test parameters can be found in Table 1, as referenced in Chen *et al.* [63].

To investigate the maximum quantum yield of PSII photochemistry (F_V_/F_M_), tea plants were dark-adapted for 30 min using a pulse-modulated Imaging-PAM M-series fluorometer (Heinz Walz GmbH, Effeltrich, Germany) with the MAXI-version [35]. Nine individual tea plants were measured for each treatment.

### Determination of photosynthetic pigments

The contents of chlorophyll and carotenoid were measured following a previous study [64]. Exactly 0.1 g of fresh tea leaves were ground into a homogenate using 100% ethanol and allowed to discolor. The absorbance values of the extracts were measured at A_665_, A_649_, and A_470_.

### Analysis of MDA, soluble sugar, soluble protein, and proline content

MDA content was determined using the MDA Kit, strictly following the manufacturer’s protocol (Solarbio Co., Ltd., Beijing, China). In brief, 0.1 g of fresh tea leaves were homogenized with 0.9 mL of extraction solution in a chilled mortar using a pestle. The homogenate was centrifuged at 8000 rpm for 10 minutes, and the resulting supernatant was collected for MDA measurement at wavelengths of 450, 532, and 600 nm.

The anthrone colorimetric method was used to determine total soluble sugar content, with glucose serving as the standard. Briefly, 0.1 g of the pulverized sample was mixed with 1 mL of distilled water and boiled for 20 minutes. Following centrifugation at 13000*g* for 10 min, 200 μL of the supernatant was combined with 1.8 mL of distilled water and 2.0 mL of a 0.14% (w/v) anthrone solution dissolved in 100% H_2_SO_4_. The mixture was boiled for an additional 20 min, and the absorbance was measured at 620 nm.

Soluble protein content was analyzed using the Coomassie brilliant blue method. In brief, 0.1 g tea leaf sample was ground and mixed with 0.9 mL of extraction solution. The mixture was centrifuged at 3000 rpm for 10 minutes, and the resulting supernatant was collected. An equal volume of distilled water was added to the supernatant, followed by 5 mL of Coomassie brilliant blue solution. The soluble protein content was then determined at 595 nm.

The proline content in tea leaf samples was determined according to the method described by Cao *et al.* [65]. The absorbance was measured at wavelength of 520 nm using a microplate reader (CYTATION3, Bio Tek).

### Determinations of ROS contents and antioxidative enzymes

Accumulations of hydrogen peroxide (H_2_O_2_) and superoxide radical (O_2_^**·−**^) were detected through staining with diaminobenzidine (DAB) and nitroblue tetrazolium (NBT) respectively as outlined by Zhao *et al.* [66]. Furthermore, the H_2_O_2_ and O_2_^**·−**^ content were also measured utilizing the commercial assay Kit (Solarbio Co., Ltd., Beijing, China, and Nanjing Jiancheng Bioengineering Institute, Nanjing, China, respectively) according to the manufacturer’s protocols. The absorption was measured at 405 nm for H_2_O_2_ and 415 nm for O_2_^**·−**^ using a microplate reader (CYTATION3, Bio Tek, USA).

Antioxidant enzymes, including SOD, CAT, POD, and APX, were determined using kits supplied by Nanjing Jiancheng Bioengineering Institute, following the manufacturer’s instructions. The absorbance of the antioxidant enzymes was determined at wavelengths 550, 405, 420, and 290 nm, respectively. AsA content was measured with a slightly modified method described by Zhang *et al.* [67]. Specifically, fresh tea leaves (0.1 g) were milled with a solution containing 1 mL absolute ethyl alcohol, l.9 mL of 5% (w/v) trichloroacetic acid, 0.5 mL of 0.03% FeCl_3_–ethanol, 0.5 mL of 0.5% H_3_PO_4_–ethanol, and 1 mL 0.5% bathophenanthroline-ethyl alcohol. After a 60-minute incubation, the liquid was determined at wavelength 534 nm. GSH content was measured using a GSH Assay Kit (Solarbio, Beijing, China) with the absorbance recorded at 412 nm. GST activity was determined following the protocol provided with a glutathione S-transferase kit (Nanjing Jiancheng Bioengineering Institute, Nanjing, China).

### Transcriptome sequencing and functional enrichment analyses

A total of 33 high-quality RNA samples from AMHA- and mock-treated tea plant was prepared for RNA-seq. RNA-seq was performed by Majorbio Biotech (Shanghai, China). Total RNA was extracted from frozen leaf samples using an MJZol total RNA extraction kit. The integrity of the extracted RNA was rigorously assessed using an RNA 6000 Nano kit and a Bioanalyzer 2100 (Agilent Technologies, CA, USA). For sequencing library construction, 1 μg RNA per sample was processed using the NEBNext Utra™ RNA Libraries Prep kit for Illumina (NEB, USA), following which cDNA synthesis, Illumina sequencing, and quality control were performed as described by Shen et al*.* [68].

The clean reads were aligned to the reference tea plant genome sequence (HD), which has the project number PRJCA003382, and is sourced from the BIG Data Center (https://bigd.big.ac.cn/), using HISAT2 *v*2.0.5 and StringTie *v*1.3.3 software [69, 70]. To quantify gene expression levels, the transcripts per kilobase of exon model per million mapped reads (TPM) value for each unigene were calculated employing RSEM software. The raw counts obtained for each unigene were analyzed using the EDSeq2 R package to identify for DEGs between AMHA- and mock-treated plants. Stringent criteria were employed for DEGs identification, including a |log_2_ (fold change) | ≥ 1 and *P* < 0.05. Furthermore, GO and KEGG pathway enrichment analyses were performed using the Majorbio cloud platform (https://www.majorbio.com).

### Time-ordered gene co-expression network (TO-GCN) construction

To construct TO-GCNs, cutoff values for TFs and non-TFs were determined. Then, co-expression networks were built based on distinct co-expression patterns, and the time-ordered levels of nodes within each network were established. Pearson’s correlation coefficients (PCCs) between TFs and non-TFs were calculated. PCCs exceeding 0.94 were deemed statistically significant at *P* < 0.05 (Table S7). Accordingly, genes with PCCs ≥ 0.94 were classified as positive co-expressors, those with PCCs falling between −0.88 and 0.94 (exclusive) as non-co-expressors, and those with PCCs < −0.88 as negative co-expressors. The TO-GCNs were visualized using Cytoscape *v*3.9.0 [71].

### *Cis*-acting elements analysis

To gain further insights into the regulatory elements within the promoter regions of DEGs in key pathways, the upstream regions (2000 bp) of flavonoid/anthocyanin, carotenoid, and AsA biosynthesis-related genes, as well as *GST* genes were extracted and analyzed using PlantCARE (http://bioinformatics.psb.ugent.be/webtools/plantcare/html/) for *cis*-elements. The results were then visualized through TBtools after manual filtering process [72].

### Quantitative real-time PCR (RT-qPCR)

Leaves from *C. sinensis* ‘Baiye 1’ plants treated with AMHA and 0.01% Tween-20 following cold treatment were collected for RNA extraction. Total RNA was isolated from 0.1 g leaf materials using an SteadyPure Plant RNA Extraction kit (Accurate Biotechnology Co., Ltd, China). First-strand cDNA was synthesized using a cDNA synthesis SuperMix for RT-qPCR. PCR amplification of key TFs was performed on a CFX96 Touch Real-Time PCR Detection System (BIO-RAD, USA) under the following thermal cycling conditions: an initial denaturation step at 95°C for 30 seconds, followed by 40 cycles of 95°C for 5 seconds and 58°C for 30 seconds. The relative gene expression level was calculated using the 2^−△△CT^ method. Three biological replicates were recorded for each assay, and the *Csβ-actin* gene serving as the internal standard. The RT-qPCR primers used in this study are detailed in Table S8.

### Gene suppression of glutathione S-transferases (*GSTs)* in tea plant

Oligodeoxynucleotide (AsODN)-mediated transient transformation was employed to induce silencing of targeted genes in cuttings of ‘Baiye 1’. Candidate AsODNs were selected using the Soligo online software with *GSTU8* (HD.04012874), *GST23*-like (HD.05G0008760), *GSTU7* (HD.10G0024130), *GSTL3* (HD.04006654), and *GSTU7.2* (HD.15G0018380) as input sequences (Table S9). Solutions of 20 μM AsODN_*GSTU8*, AsODN_*GST 23-like*, AsODN_*GSTU7*, AsODN_*GSTL3*, and AsODN_*GSTU7.2*, respectively*,* were injected into the young shoots. Tea plant injected with an equivalent volume of ddH_2_O served as controls. After 12 hours of cold stress at −4°C, the leaves were collected and stored at −80°C for further analyses.

### Cloning and expression of *CsGSTU7* in *Escherichia coli*

Total RNA was extracted following the manufacturer’s protocol of the ultrapure RNA kit. Gene-specific primers, based on the open reading frames of GSTs, were employed and are shown in Table S10. The *CsGSTU7* open reading frame was cloned into the TOPO expression vector. The expression vector (pBI121–*CsGSTU7*) containing the target gene via *BamHI* restriction sites using ClonExpress Ultra One Step Cloning Kit V3 (Vazyme), was then transformed into the *Escherichia coli* strain DH5α for expression.

### *CsGSTU7* overexpression in tea plant

Transient expression was used to analyze the functions of GSTs by overexpressing them in tea plant, due to the absence of a stable genetic transformation system. The *CsGSTU7* was inserted into the pBI121 plasmids and introduced into the host strain GV3101 (Shanghai Weidi Biotechnology Co., Ltd), with the pBI121 empty vector serving as the control. Mature leaves of ‘Baiye 1’ were injected with the bacterial solution after being washed and resuspended in MES buffer. After AMHA pretreatment and 12 hours of cold stress, each plant was sampled independently. The samples were immersed in liquid nitrogen for gene expression analysis, and plants exhibiting >50% increased expression levels compared to those with the EV plants were used in the experiment for physiological analysis.

### Statistics

Data are presented as means ± standard errors (SEs) using GraphPad Prism 8.0 software. Statistical significance was determined using SPSS software 26.0 (SPSS Inc., Chicago, IL, USA) with Student’s t-test. Asterisks (*) indicate significant differences between the AMHA-treated and mock evaluated for each time interval (^*^P < 0.05, ^**^P < 0.01, and ^***^P < 0.001).

## Supplementary Material

Web_Material_uhaf073

## Data Availability

All relevant data in this study were provided in the article and its supplementary files. The raw sequences of RNA-seq were submitted to the NCBI Sequence Read Archive under BioProject accession number PRJCA003382.
