# Supplementary material for: Plant resistance inducer AMHA enhances antioxidant capacities to promote cold tolerance by regulating the upgrade of glutathione S-transferase in tea plant
Source: Hortic Res. 2025 Mar 5;12(6):uhaf073. doi: 10.1093/hr/uhaf073 (PMC12038892; doi:10.1093/hr/uhaf073)
Supplement: Web_Material_uhaf073 [file web_material_uhaf073.zip › Supplementary figure.docx]

**Plant resistance inducer AMHA enhances the antioxidant capacities to promote cold tolerance by regulating the upgrade of glutathione S-transferase in tea plants**

Xuejin Chen ^1^, Ning Zhou ^1^, Lisha Yu ^1^, Zhaolan Han ^1^, Yanjing Guo ^1^, Salome Njeri Ndombi ^1^, Huan Zhang ^1^, Jie Jiang ^1^, Yu Duan ^1^, Zhongwei Zou ^2^, Yuanchun Ma ^1^, Xujun Zhu ^1^, Shiguo Chen ^1, *^, Wanping Fang ^1, *^

1 Tea Science Research Institute, Weed Research Laboratory, Nanjing Agricultural University, Nanjing 210095, PR. China

2 Department of Biology, Wilfrid Laurier University, Waterloo, N2L 3C5, Canada

Supporting Information

The following Supporting Information is available for this article:

Fig. S1. Effects of different concentrations of AMHA treatments in tea plants under cold stress.

Fig. S2. Effect of AMHA on the JIP-test parameters of tea plants.

Fig. S3. Quantitative PCR analysis of antioxidant enzyme SOD, APX, GST, and GR genes expression at the indicated times in AMHA-treated plants compared with mock.

Fig. S4. Gene expression profiles at the indicated times in AMHA-treated plants compared with mock.

Fig. S5. Gene ontology (GO) enrichment analysis based on ElasticSearch (ES) ranking derived from differential expression analysis. GO, gene ontology.

Fig. S6. Effects of AMHA on carotenoid metabolic pathways in tea plants.

Fig. S7 Analysis of cis-elements in the promoter region of flavonoid/anthocyanin, carotenoid, and AsA biosynthesis biosynthesis genes, as well as GSTs in tea plants. Analysis of *cis*-elements in the promoter region 2,000 bp upstream of the start codon. The same shape represents the same type of cis-element.

Fig. S8 Effects of AMHA on hub GST genes in tea plants under cold-stress conditions.

Fig. S9 Measurement of CAT activities of the *CsGSTU7* under room- and low-temperature conditions.

Fig. S10 The experimental procedure. R indicates normal cultivation stage at 2 d after cold-stress exposure.

Table S1. Formulae and explanations of the technical data of the OJIP curves and the selected JIP-test parameters used in this study.

Table S2. Processed RNA-seq data for 33 samples.

Table S3. The GO term enrichment for DEGs at each time point.

Table S4. List of enriched GO terms in the biological process category of the different expression genes at the indicated times in AMHA-treated plants compared with mock.

Table S5. The KEGG term enrichment for DEGs at each time point.

Table S6. List of enriched KEGG terms in the biological process category of the different expression genes at the indicated times in AMHA-treated plants compared with mock.

Table S7. (node pairs and PCC values) used for re-generation of the TO-GCN.

Table S8. Fluorescent quantitative primers of key transcriptional factors in tea plant.

Table S9. Antisense oligonucleotide sequences used in these studies.

Table S10. Primers used to construct the *CsGSTU7* expression vector


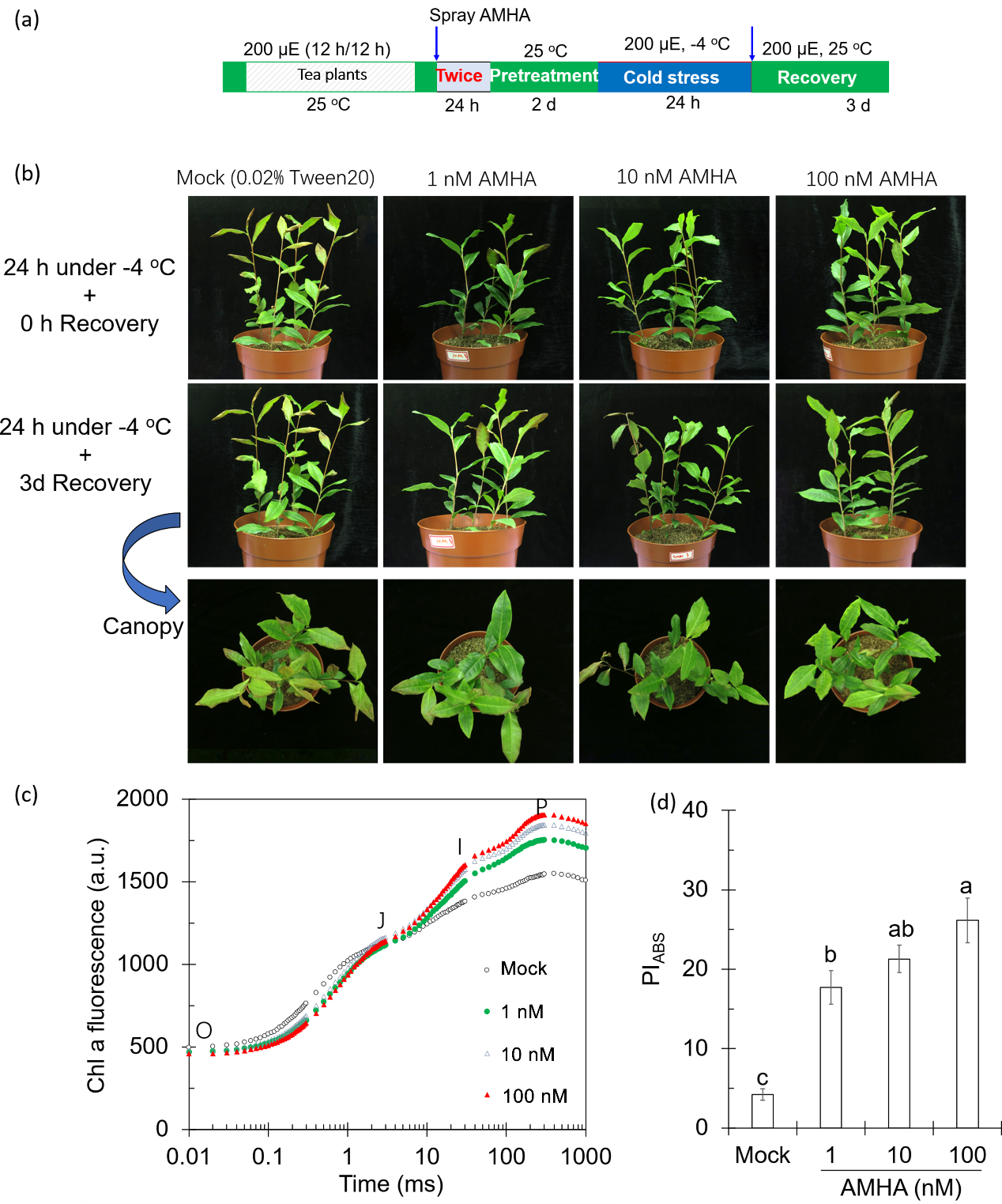


**Fig. S1** **Effects of different concentrations of AMHA treatments in tea plants under cold stress.** (a) Experimental flow chart. (b) Effect of AMHA treatment on the morphology characteristics of tea plants under cold stress (treated at −4℃ for 24 h and recovery at room temperature for 3 d). (c) Effect of AMHA pretreatment on the FV/FM of tea plant leaves under cold stress. (d) Effect of AMHA pretreatment on the PIABS of tea plant leaves under cold stress. Different letters indicate statistically significant differences (p < 0.05) based on one-way analysis of variance (ANOVA).

**
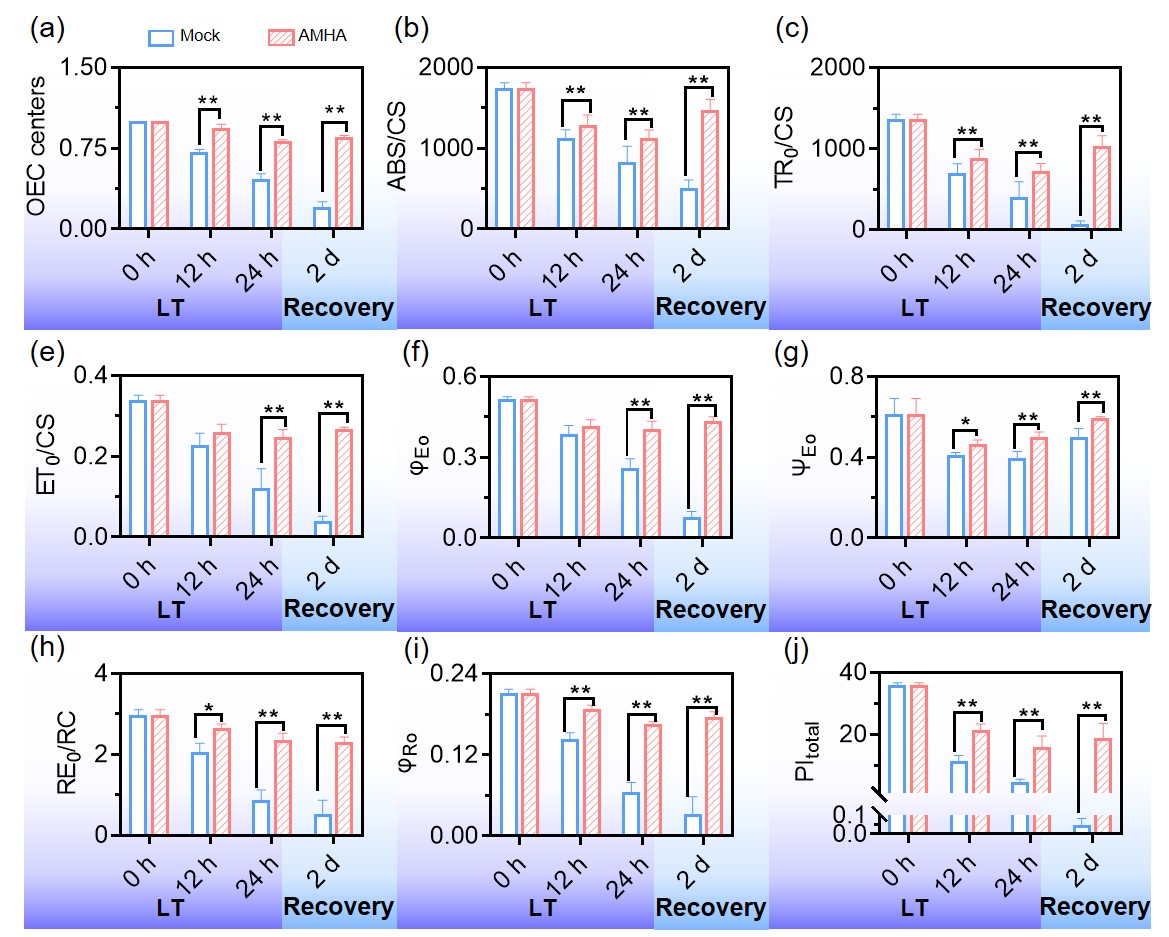
**

**Fig. S2 Effect of AMHA on the JIP-test parameters of tea plants.** (a) The fraction of oxygen-evolving complex (OEC) centers. (b) The chlorophyll concentration per excited leaf cross-section (ABS/CS). (c) Trapping energy flux per excited leaf cross-section (TR_0_/CS). (d) The QA-reducing RCs per excited leaf cross-section (RC/CS). (e) electron transport flux per CS (ET_0_/CS) (f) quantum yield of electron transport (at t = 0) (φ_Eo_). (g)probability (at t = 0) that a trapped exciton moves an electron into the electron transport chain beyond QA (Ψ_EO_). (h) Electron flux reducing end electron acceptors at the PSI acceptor side per RC(RE_0_/RC). (i) Quantum yield for reduction of the end electron acceptors at the PSI acceptor side (φ_RO_). (j) Performance index of energy conservation from photons absorbed by PSII to PSI terminal receptors (PI_total_). Data represent means ± Standard deviation (SD) of three replicates. Asterisks indicate significant differences relative to controls (Student’s *t*-test: ^*^*p* < 0.05, ^**^*p* < 0.01, and ^***^*p* < 0.001).

**
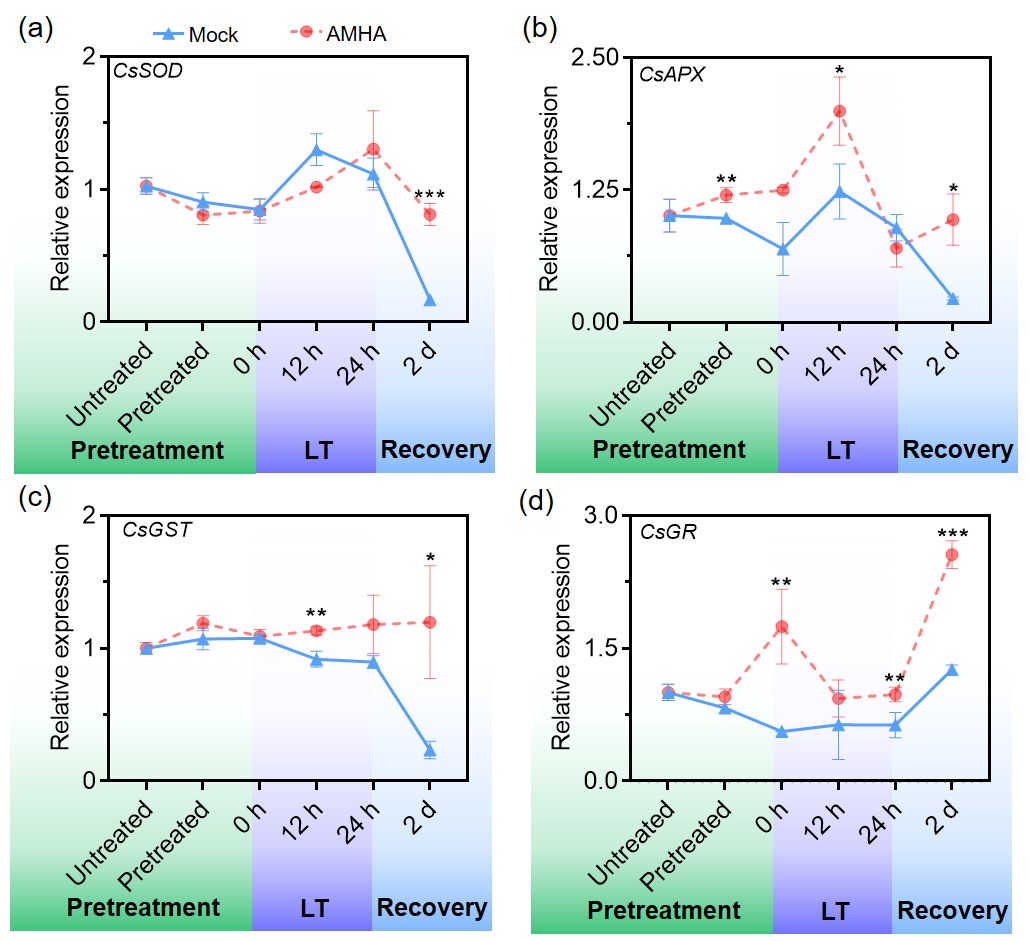
**

**Fig. S3** **Quantitative PCR analysis of antioxidant enzyme *CsSOD*, *CsAPX*,** ***CsGST*, and *CsGR* genes expression at the indicated times in AMHA-treated plants compared with mock.** (a–d) The expression levels of *CsSOD* (a), *CsAPX* (b), *CsGST* (c). and *CsGR* (d). Data represent means ± standard deviations (SDs) of three biological replicates. Asterisks indicate significant differences relative to controls (Student’s *t*-test: ^*^*p* < 0.05, ^**^*p* < 0.01, and ^***^*p* < 0.001).


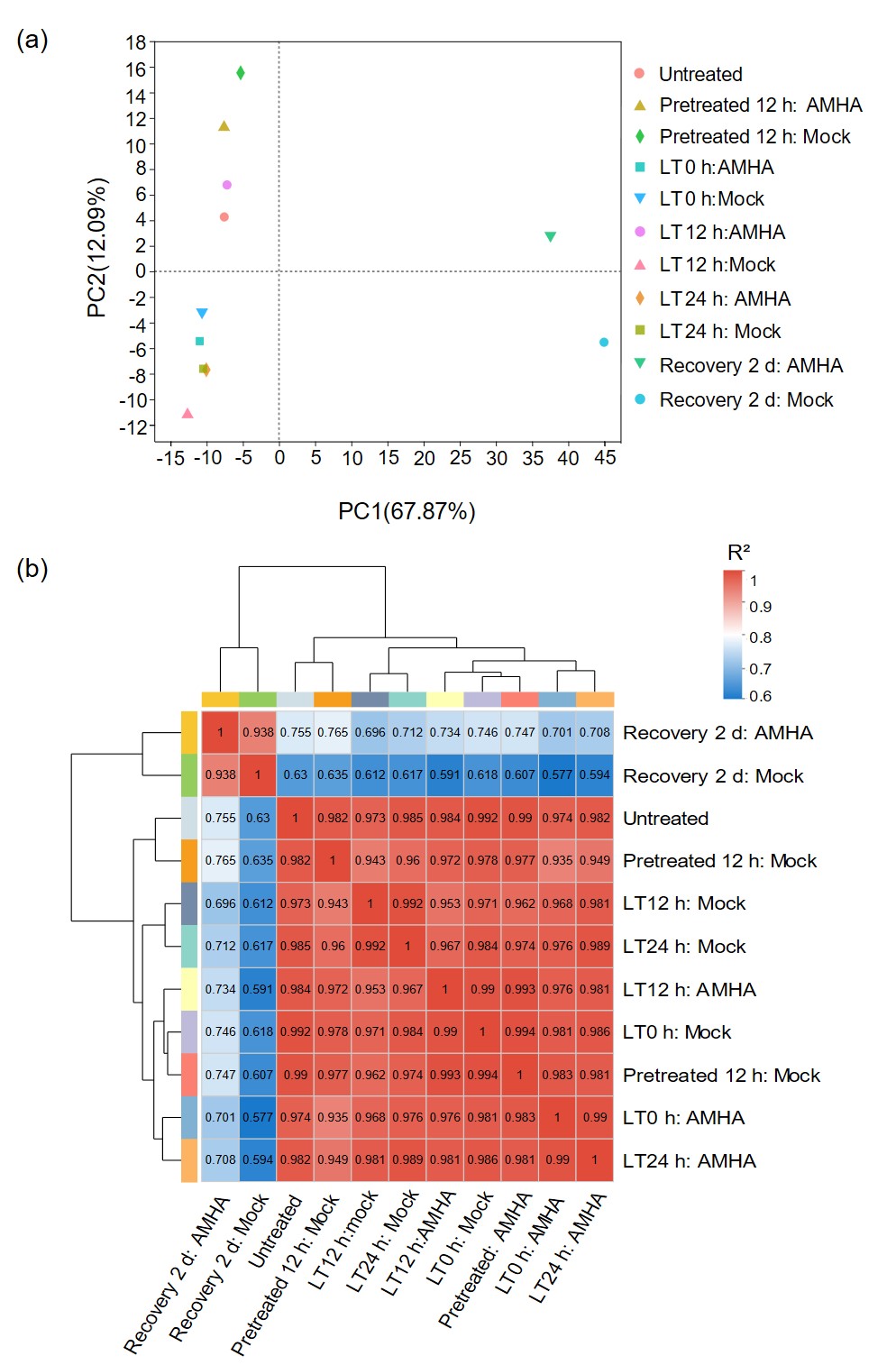


**Fig. S4 Gene expression profiles at the indicated times in AMHA-treated plants compared with mock.** (a)Principal component analysis (PCA). (b) Spearman’s correlation coefficient.


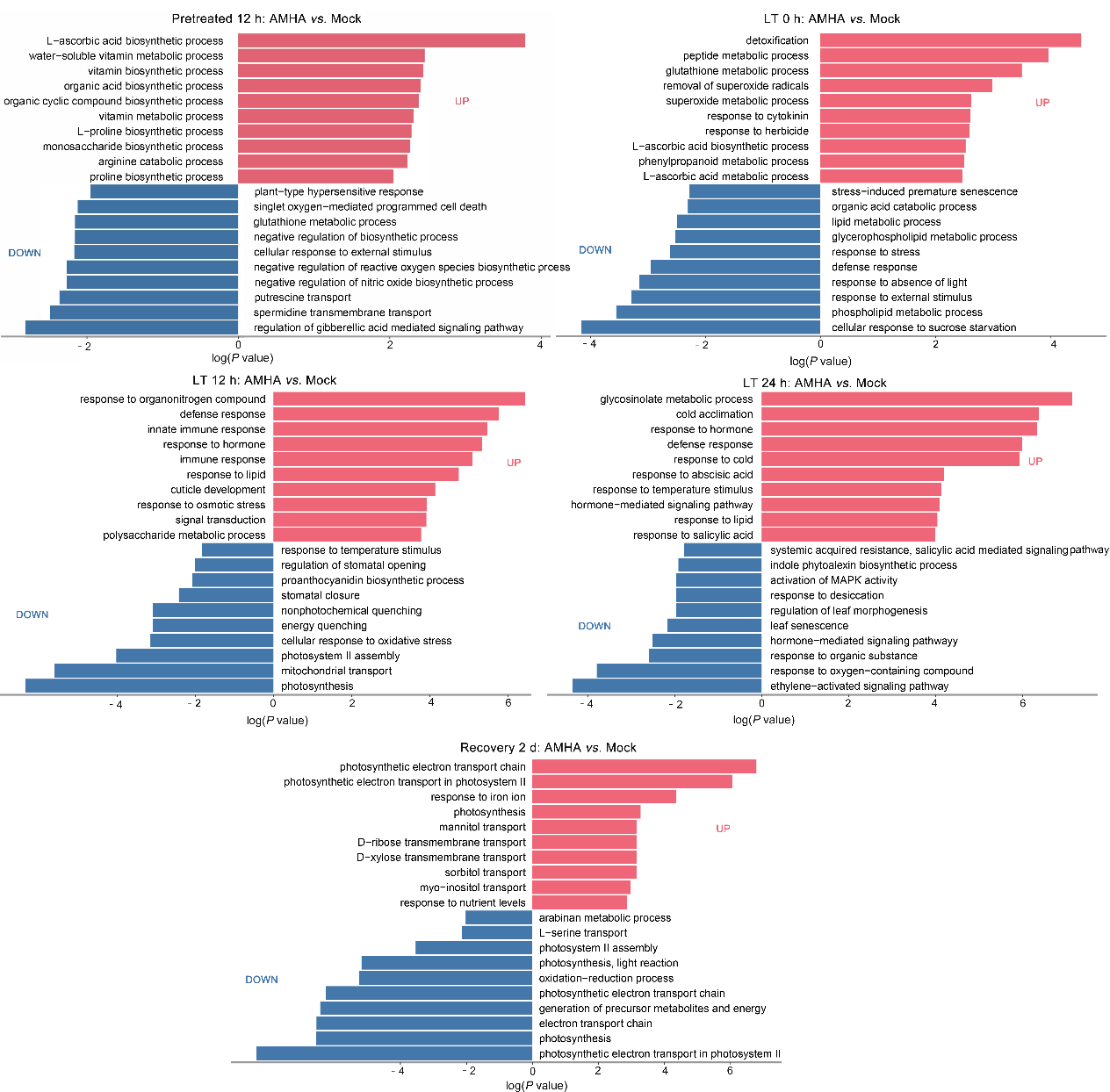


**Fig. S5** **Gene ontology (GO) enrichment analysis based on** **ElasticSearch (ES) ranking derived from differential expression analysis.** Gene ontology (GO) enrichment analysis was performed to identify the differential genes that were elevated in AMHA-treated tea plants compared to the mock at various stages, including at 12 h of pretreatment (a), 0 h of cold stress (b), 12 h of cold stress (c), 24 h of cold stress (d), and 2 d of recovery (e). For more detailed information about the GO term pathway, please refer to Table S3-S4.


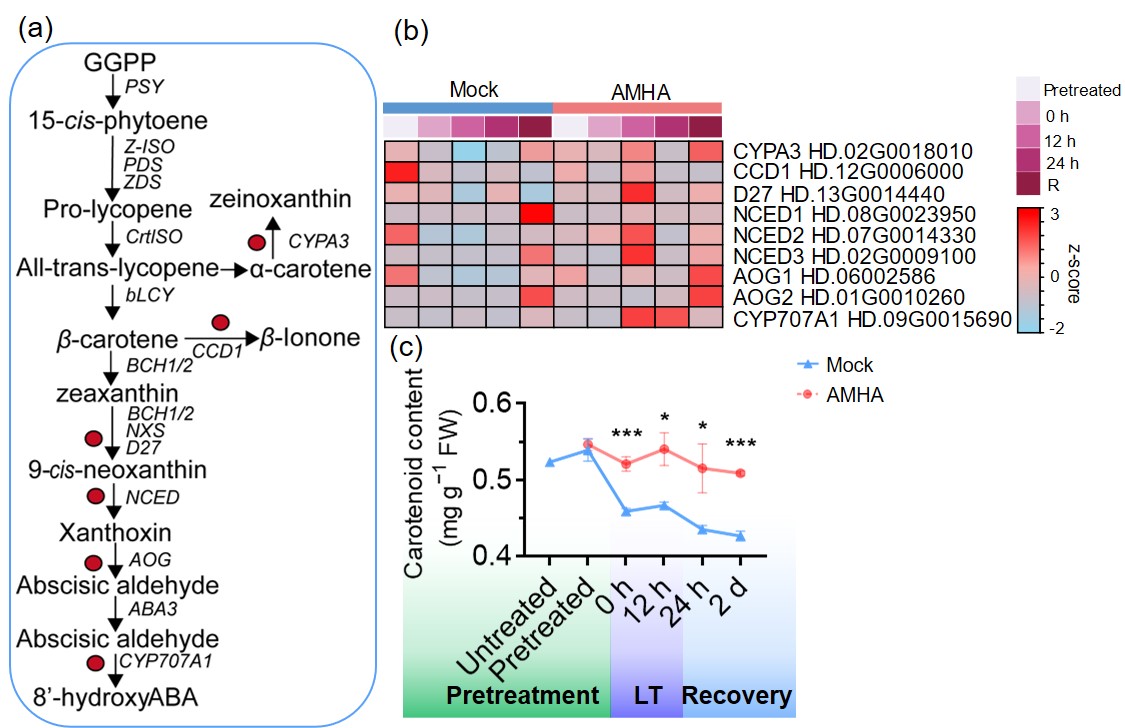


**Fig. S6 Effects of AMHA on carotenoid metabolic pathways in tea plants.** (a) and (b) The expression patterns of key genes in the carotenoid metabolism pathway. *PSY*, phytoene synthase; *Z-ISO*, ζ-carotene isomerase; *PDS*, phytoene desaturase; ZDS, ζ-carotene desaturase; *CRTISO*, carotenoid isomerase; *eLCY*, lycopene ε-cyclase; *bLCY,* lycopene β-cyclase; *CHY*, β-carotene hydroxylase; *CYPA3*, cytochrome P450-type monooxygenase; *NXS*, neoxanthin synthase; *CCD*, carotenoid cleavage dioxygenase; *NCED*, 9-cis-epoxy carotenoid dioxygenase; *CYP707A1*, abscisic acid-8′-hydroxylase. (c) The contents of total carotenoid. Data represent means ± SDs of three biological replicates. Asterisks indicate significant differences relative to controls (Student’s *t*-test: ^*^*p* < 0.05, ^**^*p* < 0.01, and ^***^*p* < 0.001).


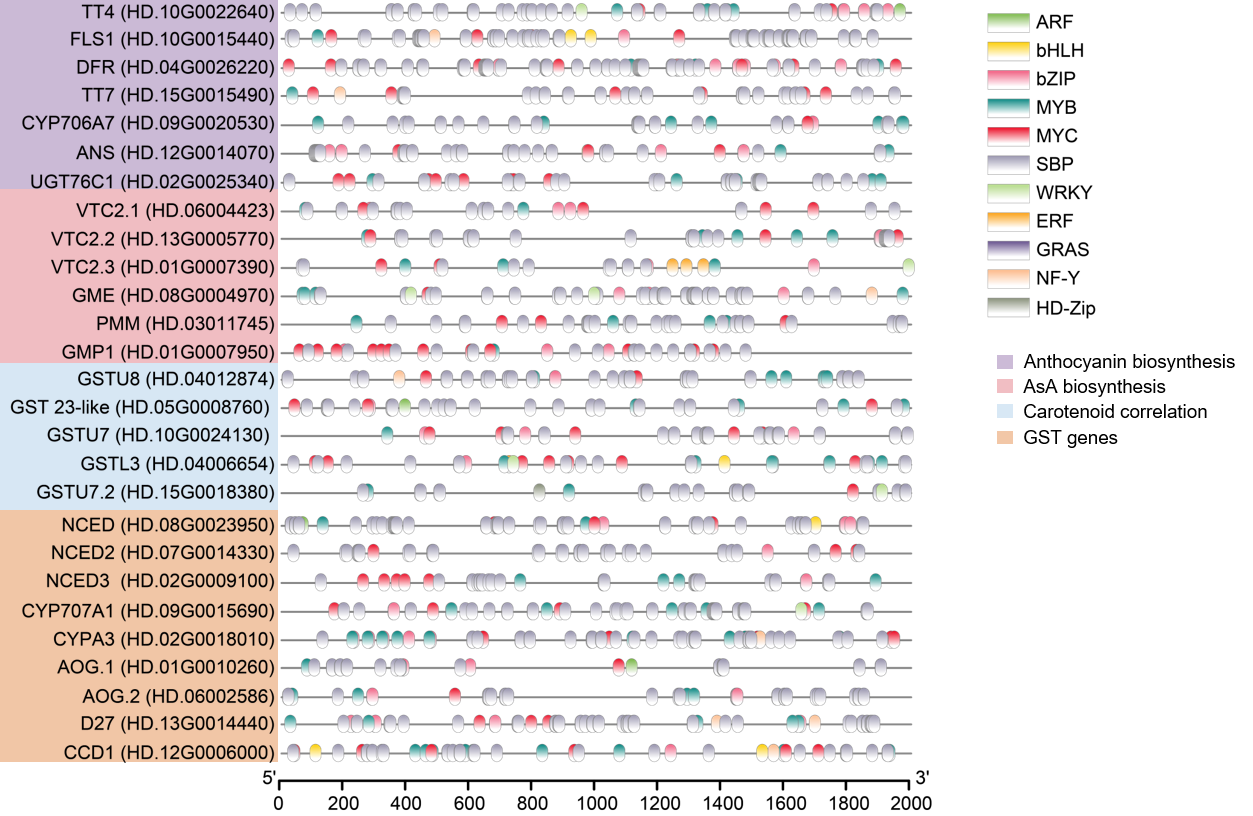


**Fig. S7 Analysis of cis-elements in the promoter region of flavonoid/anthocyanin, carotenoid, and AsA biosynthesis biosynthesis genes, as well as GSTs in tea plants. Analysis of cis-elements in the promoter region 2,000 bp upstream of the start codon. The same shape represents the same type of cis-element.**


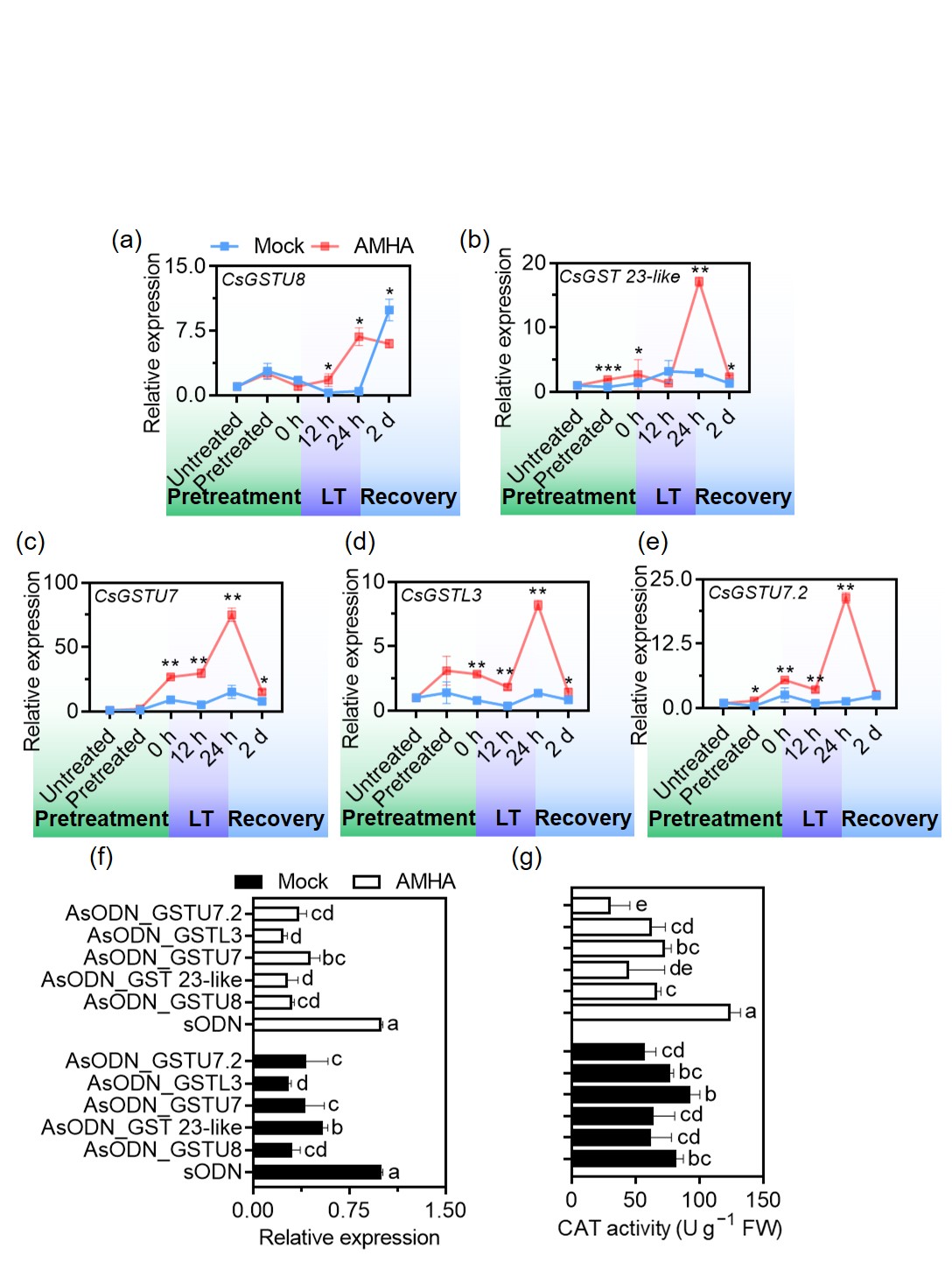


**Fig. S8 Effects of AMHA on hub *GST* genes of tea plants under cold-stress conditions.** (a) The expression patterns of *GSTs* in plants receiving AMHA treatments. The x-axis indicates the genes and treatment. The y-axis indicates the average transcripts per kilobase of the exon model per million mapped reads. (b) Quantitative PCR analysis of *GSTU8*, *GST 23-like*, *GSTU7*, *GSTL3*, and *GSTU7.2* expressions in control (sODN) and their silenced (AsODN) tea leaves under cold stress 12h. (c) The Changes of CAT of sODN and AsODN_GSTs in AMHA or mock-treated plants. Data are means ± SEs of atleast 3 biological replicates. Different letters for each binding site represent significant differences (*p* < 0.05, one-way ANOVA).


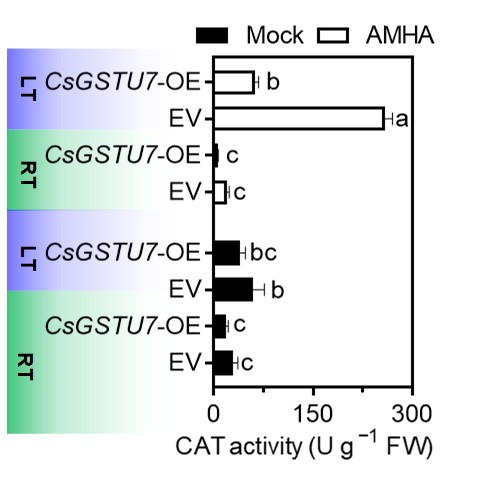


**Fig. S9 Measurement of CAT activities of the *CsGSTU7* under room- and low-temperature conditions**. The values are presented as the means ± SDs of three biological experiments.


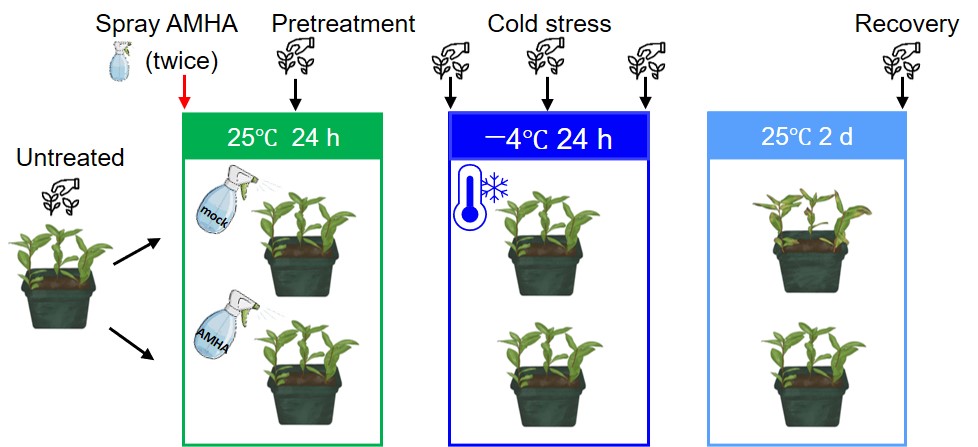


**Fig. S10 The experimental procedure. R indicates normal cultivation stage at 2 d after cold-stress exposure.**

**Table S1.** Formulae and explanation of the technical data of OJIP curves and the selected JIP-test parameters used in this study ^a^

| **Technical fluorescence parameters** | | |  |
| --- | --- | --- | --- |
| F_t_ | | fluorescence at time t after onset of actinic illumination | |
| F_O_ ≅ F_20μs_ | | minimal fluorescence, when all PSII RCs are open | |
| F_L_ ≡ F_150μs_ | | fluorescence intensity at the L-step (150 μs) of OJIP | |
| F_K_ ≡ F_300μs_ | | fluorescence intensity at the K-step (300 μs) of OJIP | |
| F_J_ ≡ F_2ms_ | | fluorescence intensity at the J-step (2 ms) of OJIP | |
| F_I_ ≡ F_30ms_ | | fluorescence intensity at the I-step (30 ms) of OJIP | |
| F_P_ (= F_M_) | | maximal recorded fluorescence intensity, at the peak P of OJIP | |
| F_v_ ≡ F_t_ – F_O_ | | variable fluorescence at time t | |
| F_V_ ≡ F_M_ – F_O_ | | maximal variable fluorescence | |
| **Quantum efficiencies or flux ratios** | | |  |
| ϕ_Eo_ = PHI(E_0_) = ET_0_/ABS = (1– F_O_/F_M_) (1–V_J_) | quantum yield for electron transport (ET) | |  |
| ψ_Eo_ = PSI_0_ = ET_0_/TR_0_ = (1–V_J_) | probability that an electron moves further than Q_A_^-^ | |  |
| ϕ_Ro_ = RE_0_/ABS = ϕ_Po_. ψ_Eo_. δ_Ro_  = ϕ_Po_. (1–V_I_) | quantum yield for reduction of the end electron acceptors at the PSI acceptor side (RE) | |  |
| **Phenomenological energy fluxes (per excited leaf cross-section-CS)** | | |  |
| ABS/CS = Chl/CS | | absorption flux per CS |  |
| TR_0_/CS = ϕ_Po_. (ABS/CS) | | trapped energy flux per CS |  |
| ET_0_/CS = φ_Po_. ψ_Εo_. (ABS/CS) | | electron transport flux per CS |  |
| RE_0_/RC = M_0_ (1/V_J_) (1 – V_I_) | | electron flux reducing end electron acceptors at the PSI acceptor side |  |
| **Density of RCs** | | |  |
| Q_A_-reducing centers  = (RC/RC_reference_). (ABS/ABS_reference_)  = $\frac{\text{(RC/CS)}\text{treatment}}{\text{(RC/CS)}\text{control}}\text{ × }\frac{\text{(ABS/CS)}\text{treatment}}{\text{(ABS/CS)}\text{control}}$ | | The fraction of Q_A_-redcuing reaction centers |  |
| **Performance indexes** | | |  |
| $PI_{ABS}$≡ $\frac{\gamma_{\text{RC}}}{1-\gamma_{\text{RC}}}\bullet\text{ }\frac{\phi_{\text{Po}}}{1-\phi_{\text{Po}}}\text{ }\bullet\text{ }\frac{\psi_{\text{Eo}}}{1-\psi_{\text{Eo}}}$  PI_total_ ≡ __ | | performance index (potential) for energy conservation from photons absorbed by PSII to the reduction of intersystem electron acceptors  performance index (potential) for energy conservation from photons absorbed by PSII to the reduction of PSI end acceptors |  |

^a^ Subscript “0” (or “o” when written after another subscript) indicates that the parameter refers to the onset of illumination when all RCs are assumed to be open.
