# Supplementary figures and images for: Plant resistance inducer AMHA enhances antioxidant capacities to promote cold tolerance by regulating the upgrade of glutathione S-transferase in tea plant
Source: Hortic Res. 2025 Mar 5;12(6):uhaf073. doi: 10.1093/hr/uhaf073 (PMC12038892; doi:10.1093/hr/uhaf073)

Spray AMHA  
(twice)

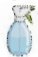

Pretreatment

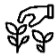

Cold stress

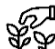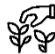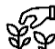

Recovery

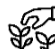

25°C 24 h

-4°C 24 h

25°C 2 d

Untreated

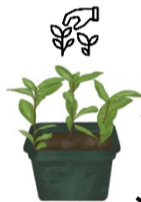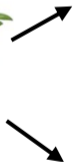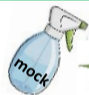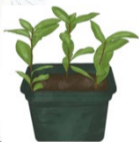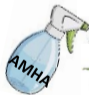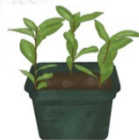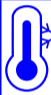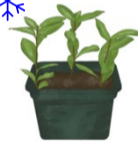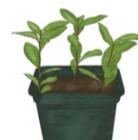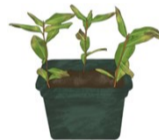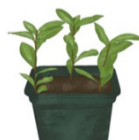

Supplement: Web_Material_uhaf073 [file web_material_uhaf073.zip › Fig. S10.pdf]

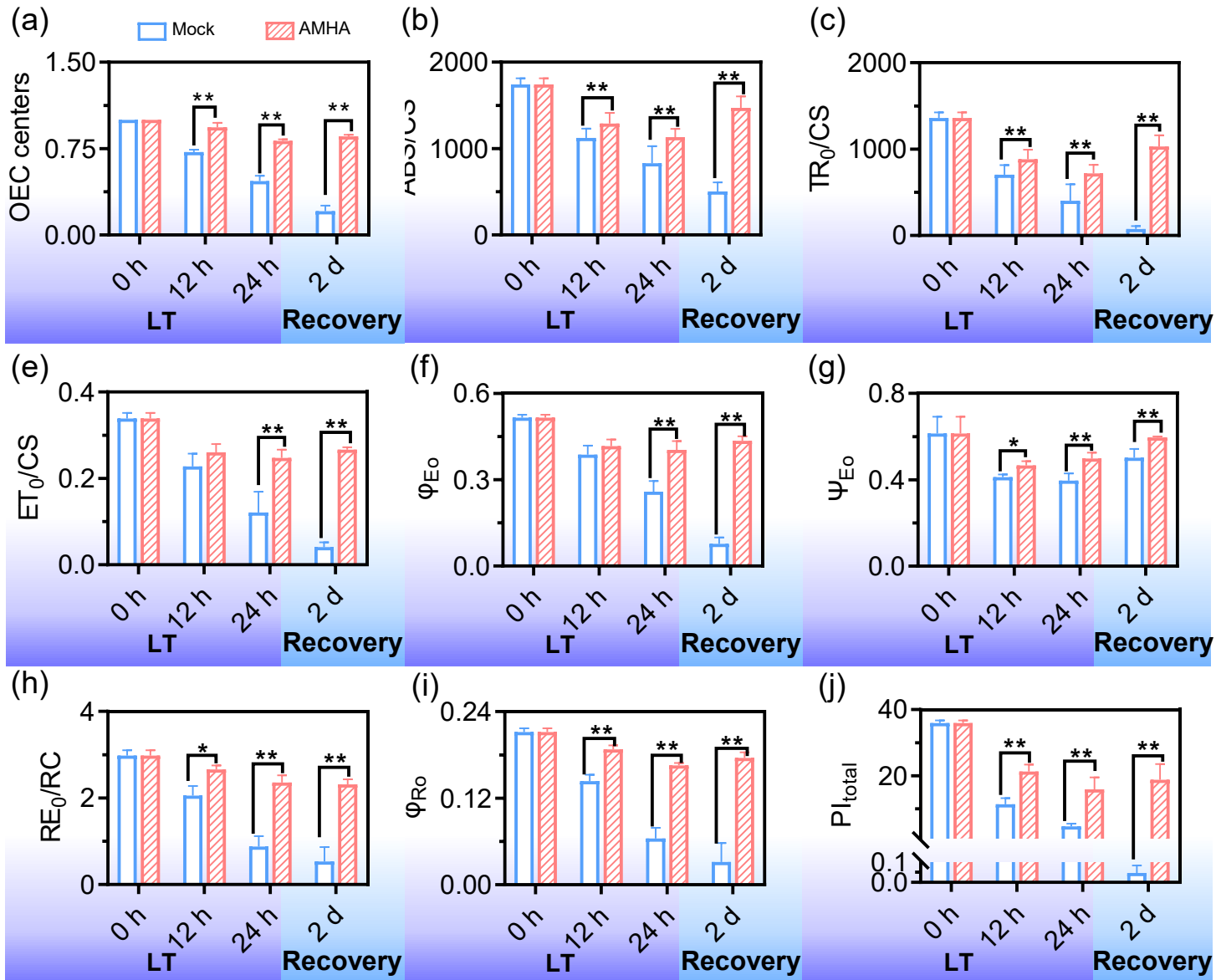

Supplement: Web_Material_uhaf073 [file web_material_uhaf073.zip › Fig. S2.pdf]

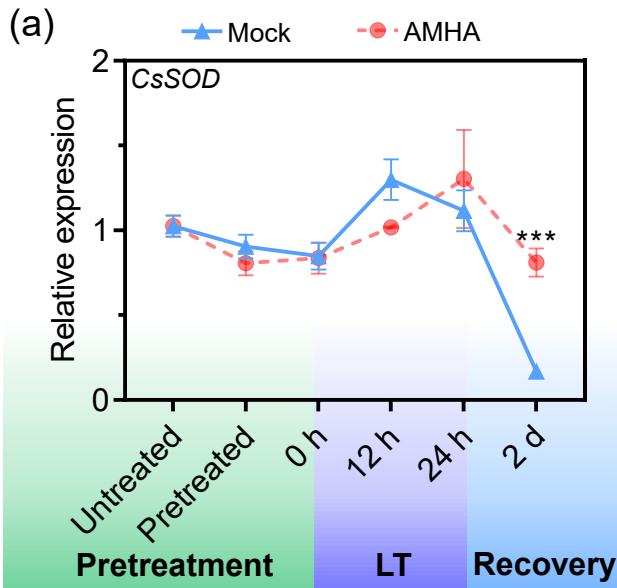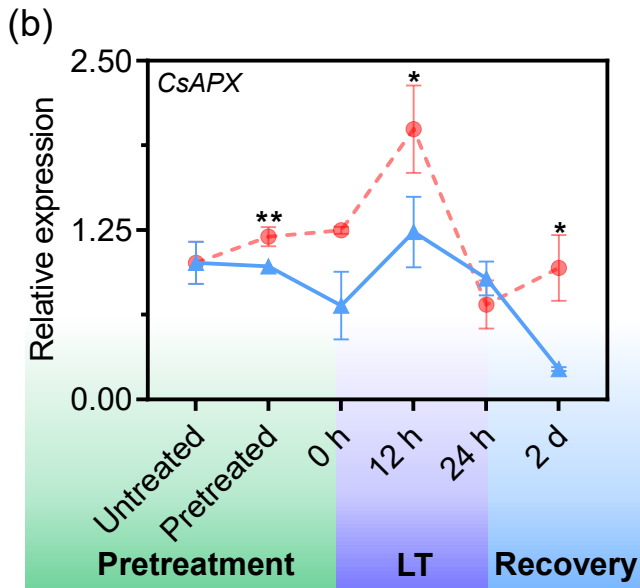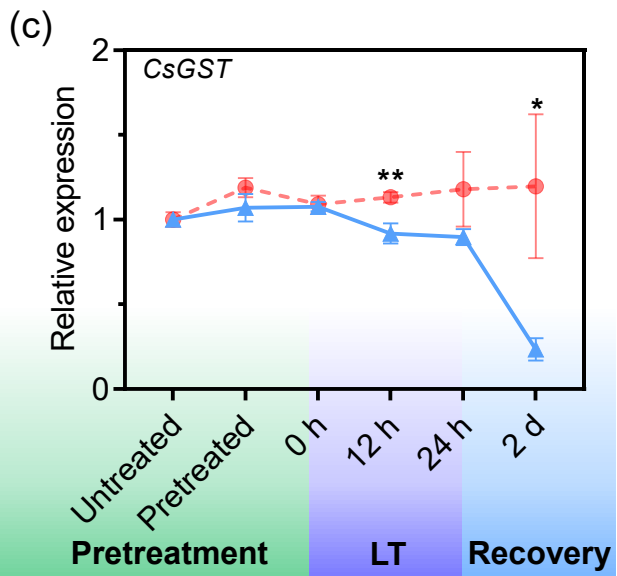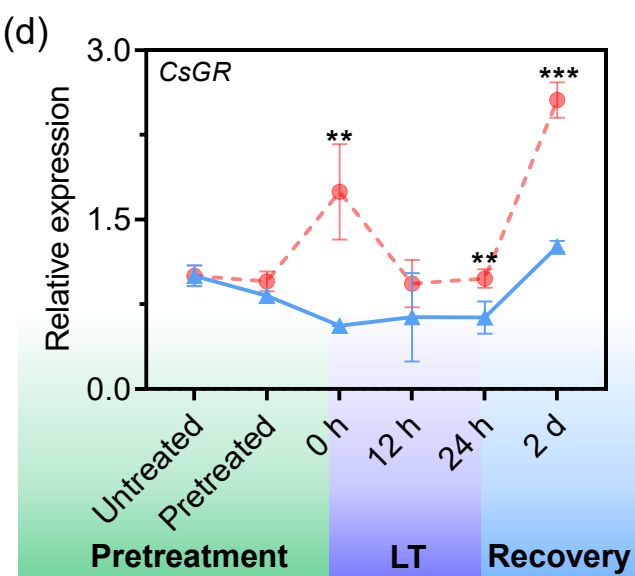

Supplement: Web_Material_uhaf073 [file web_material_uhaf073.zip › Fig. S3.pdf]

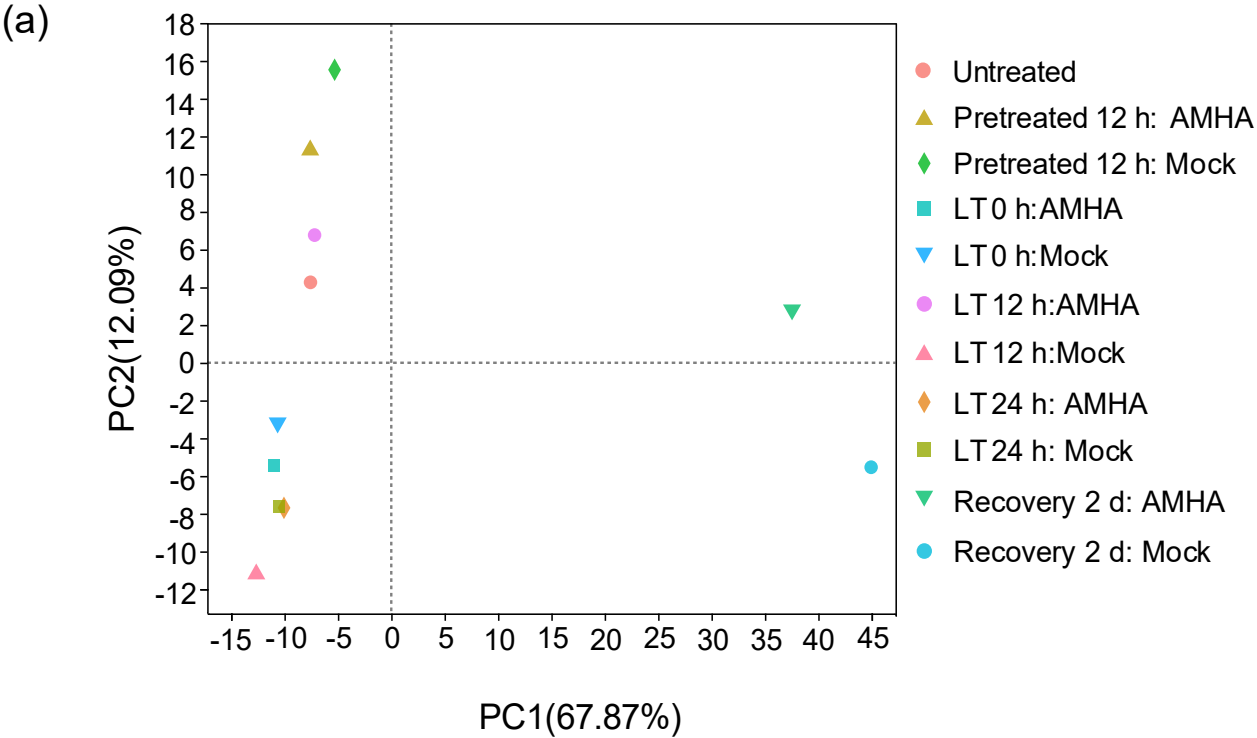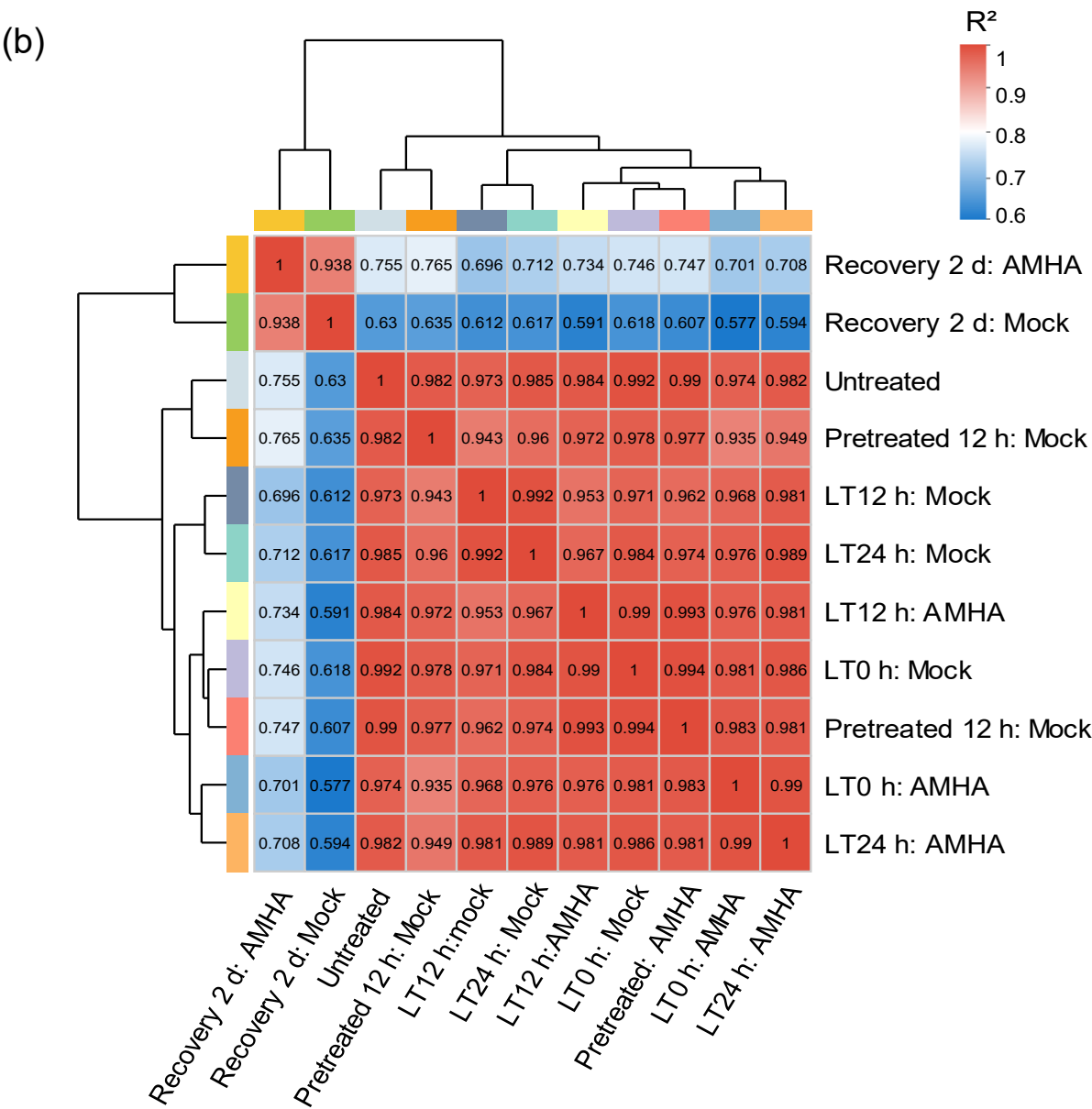

Supplement: Web_Material_uhaf073 [file web_material_uhaf073.zip › Fig. S4.pdf]

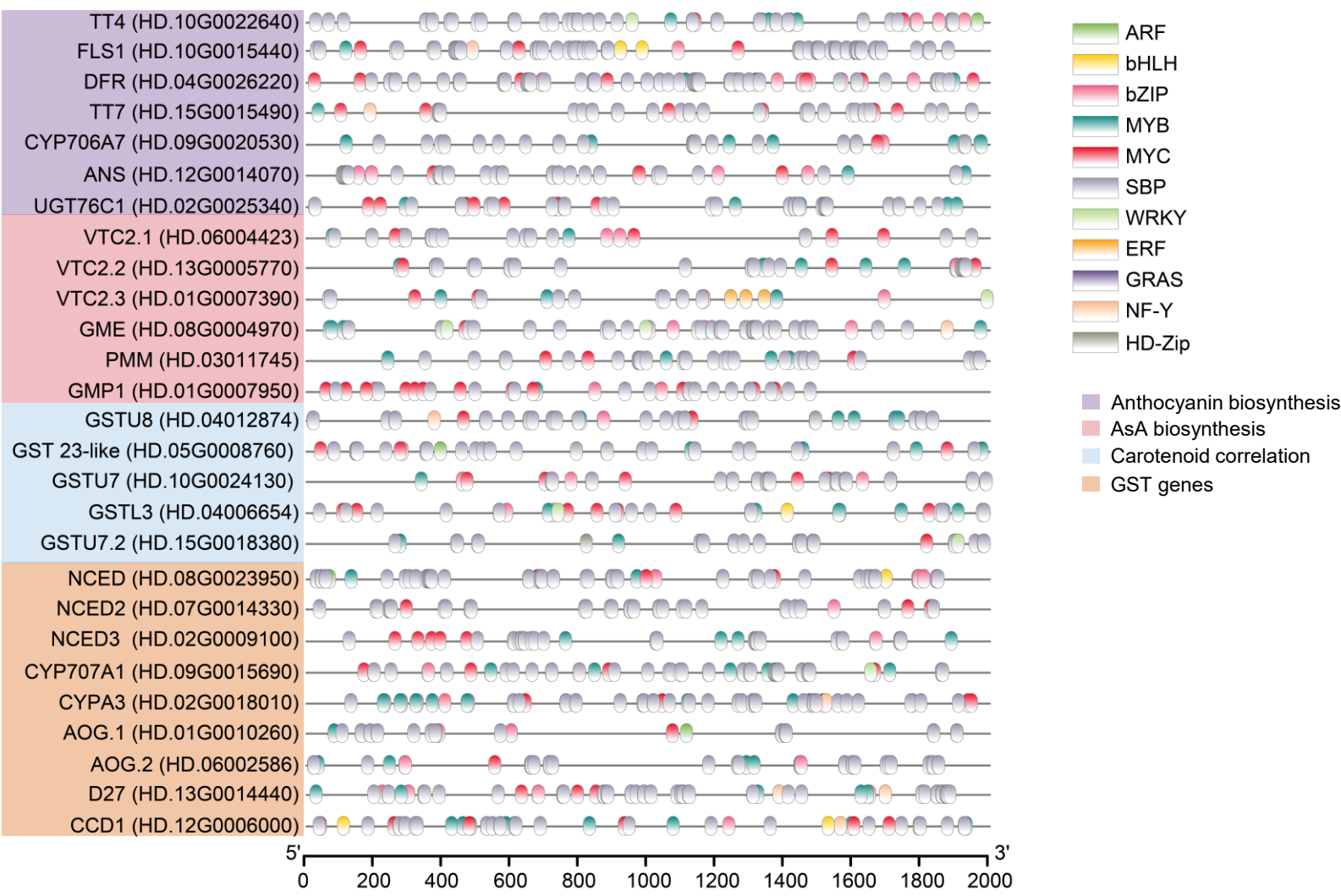

Supplement: Web_Material_uhaf073 [file web_material_uhaf073.zip › Fig. S7.pdf]

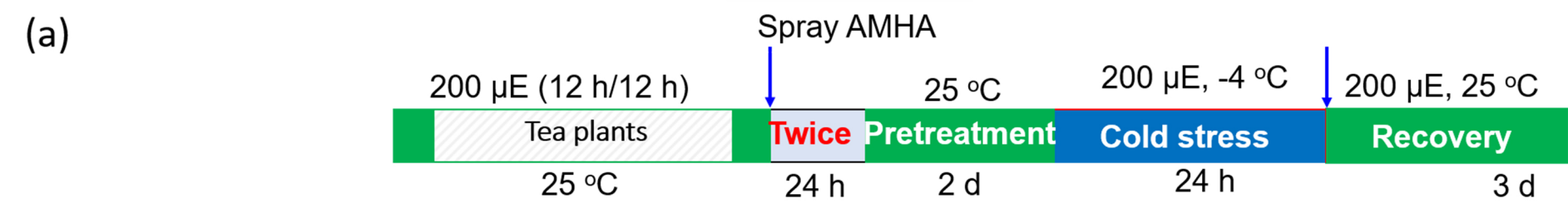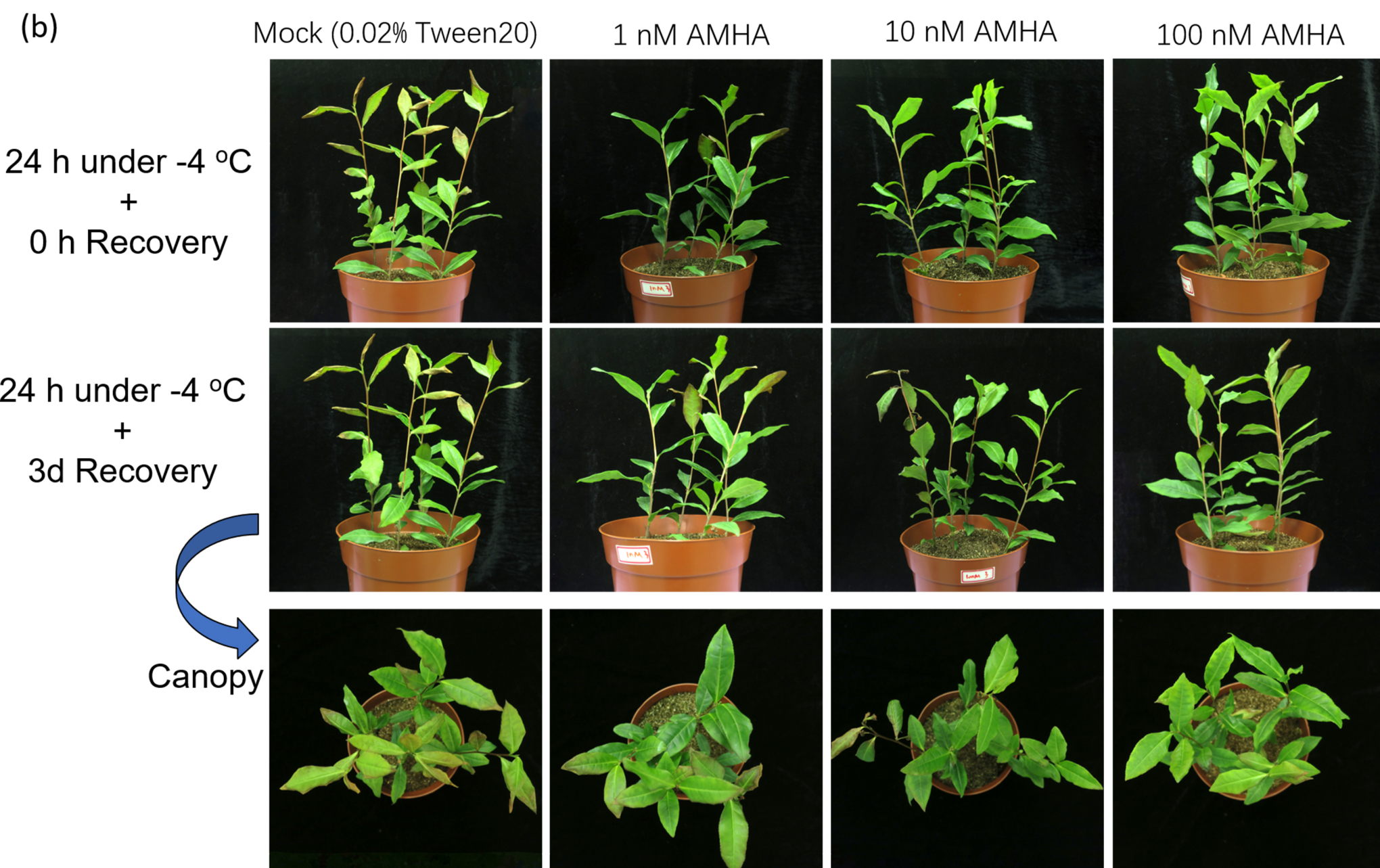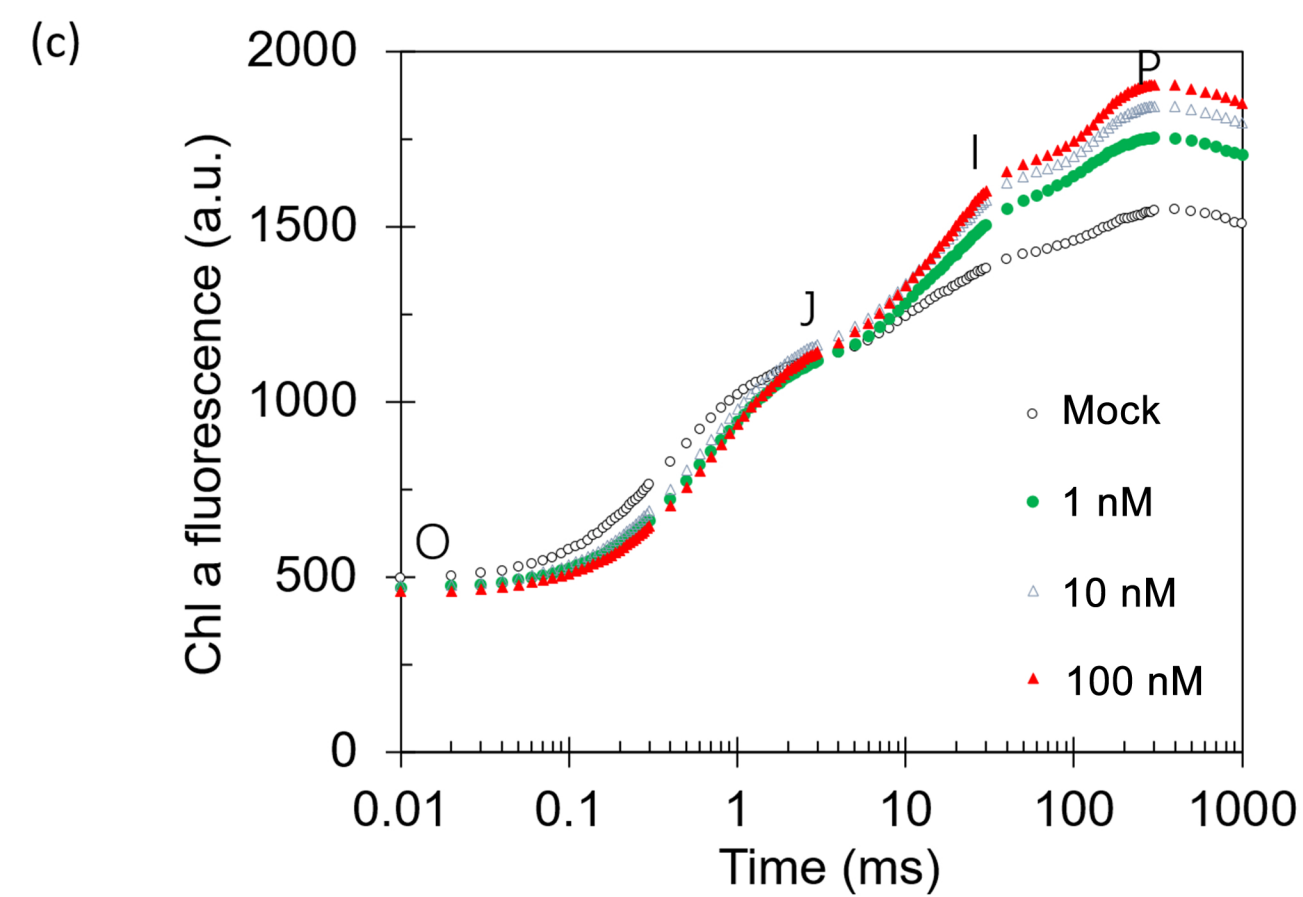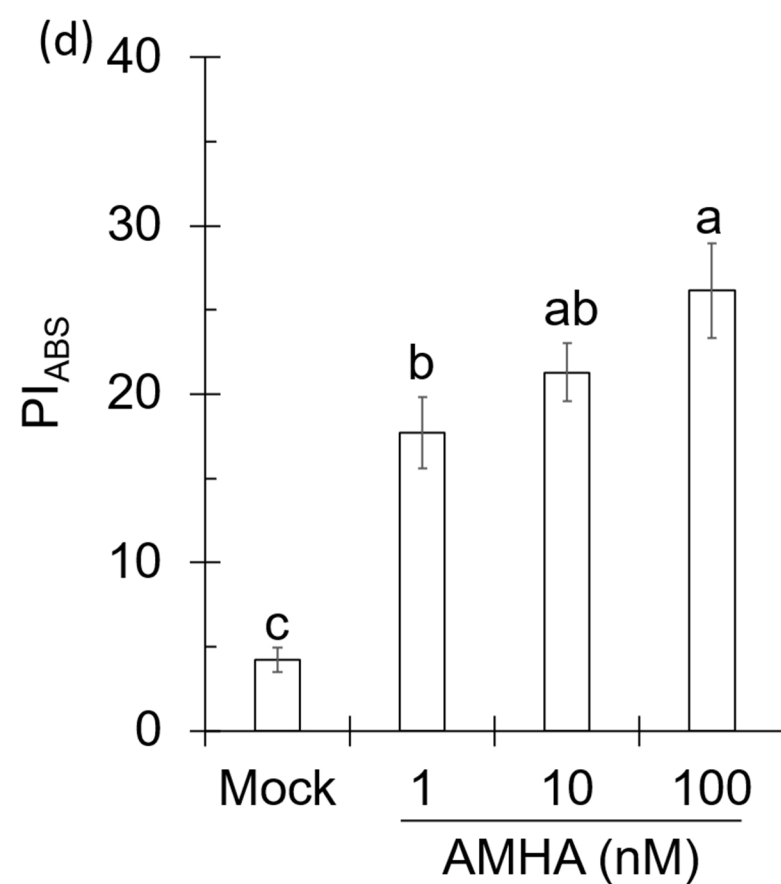

Supplement: Web_Material_uhaf073 [file web_material_uhaf073.zip › S1.pdf]

Pretreated 12 h: AMHA vs. Mock

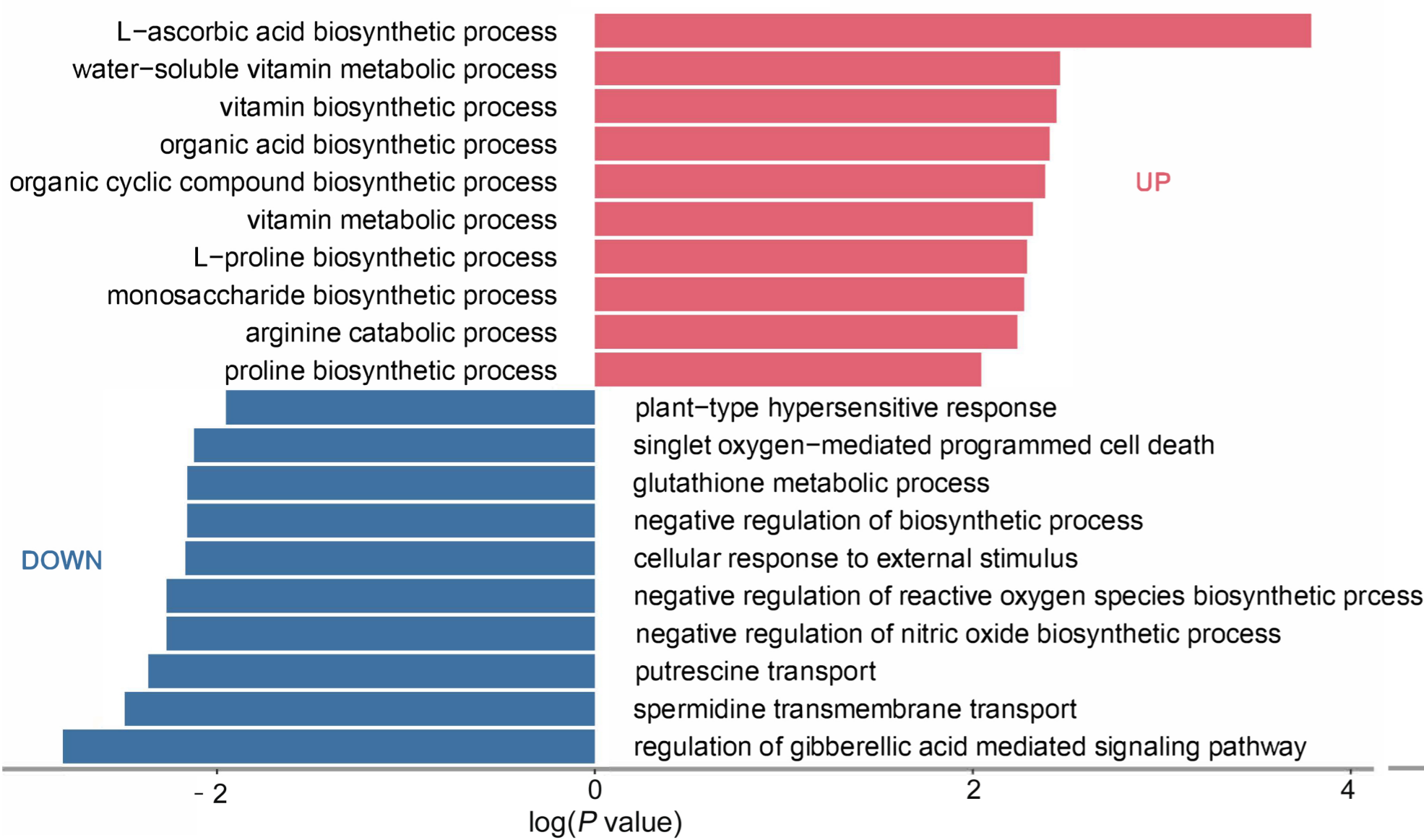

LT 0 h: AMHA vs. Mock

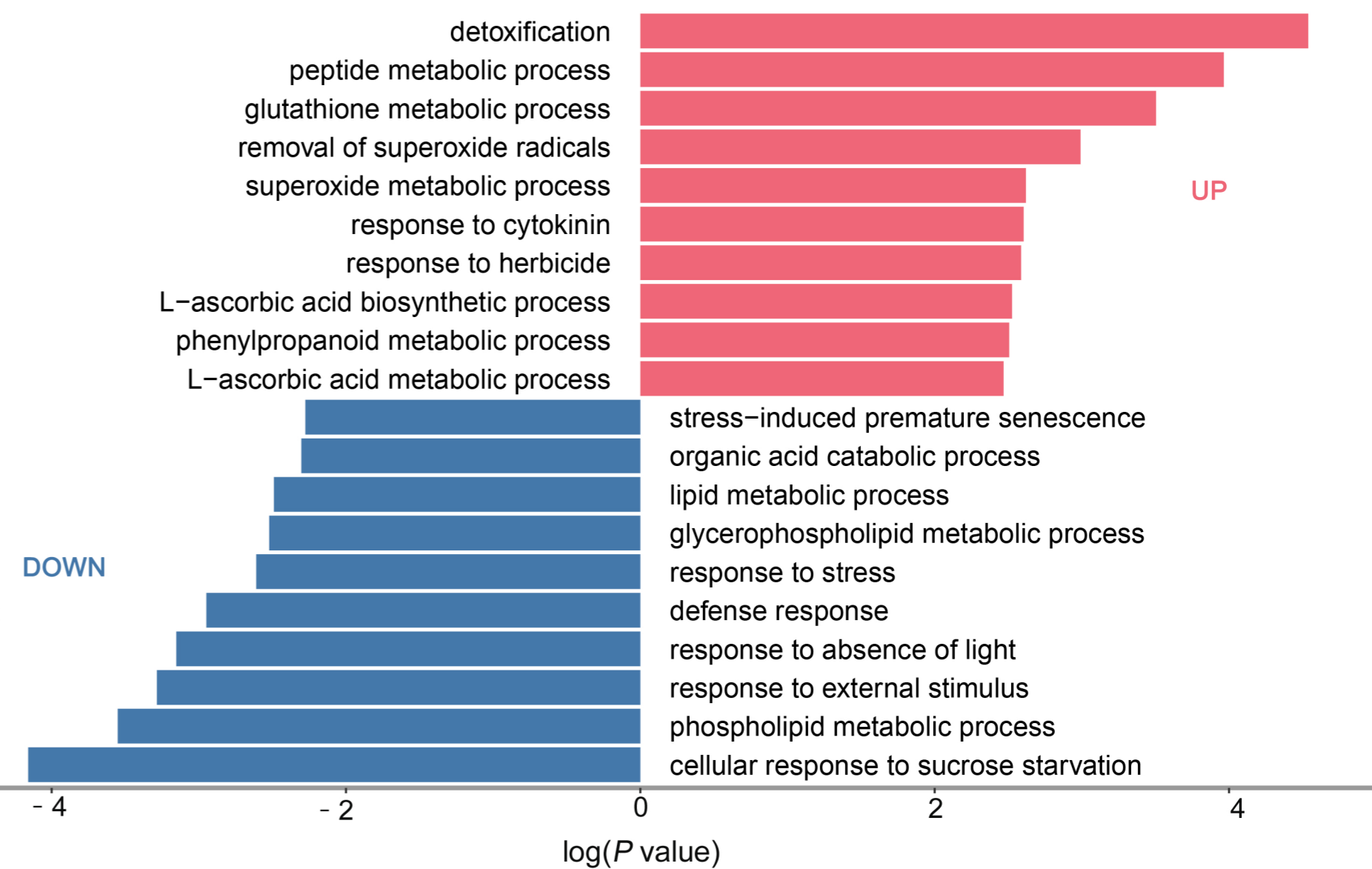

LT 12 h: AMHA vs. Mock

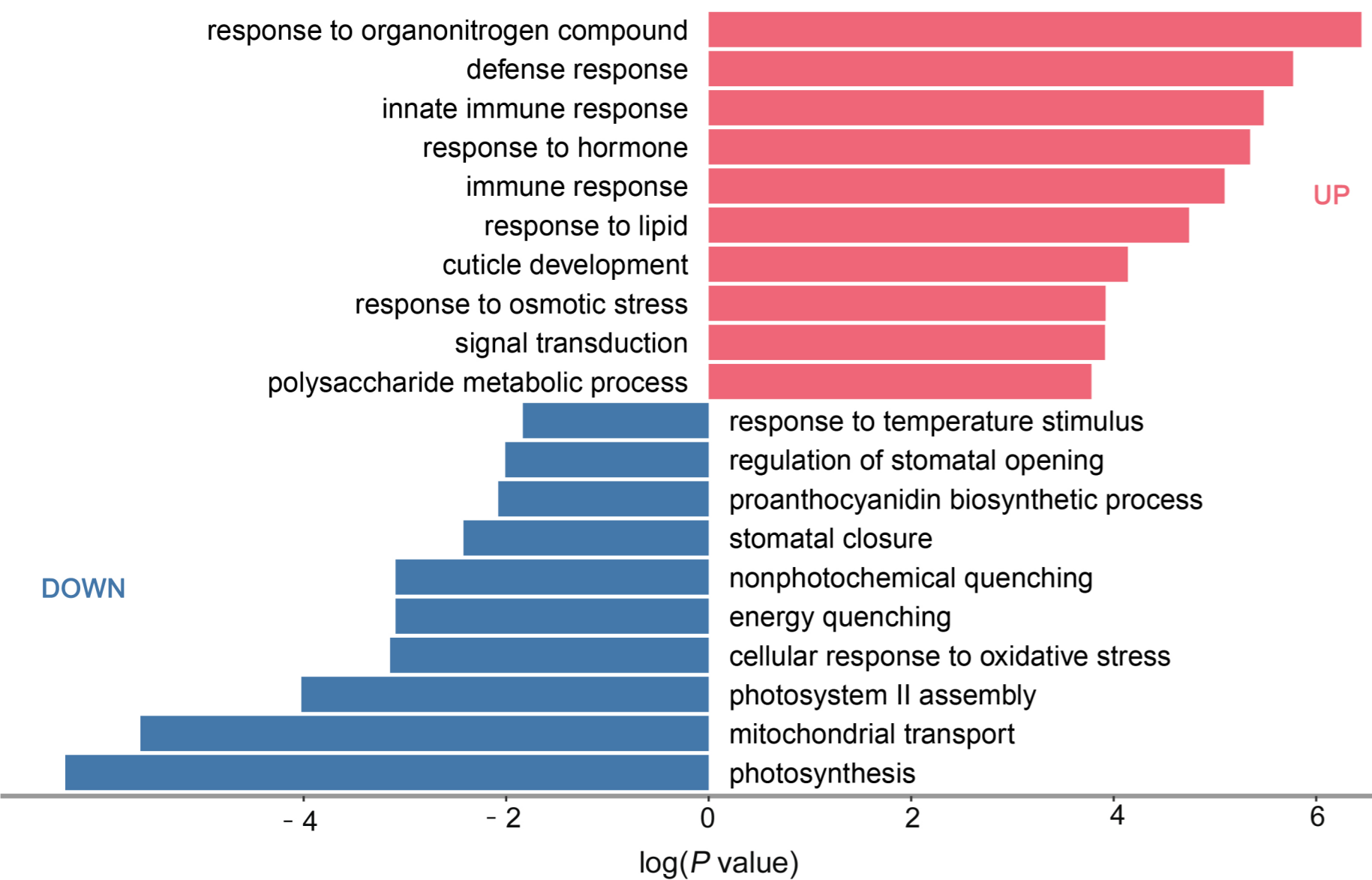

LT 24 h: AMHA vs. Mock

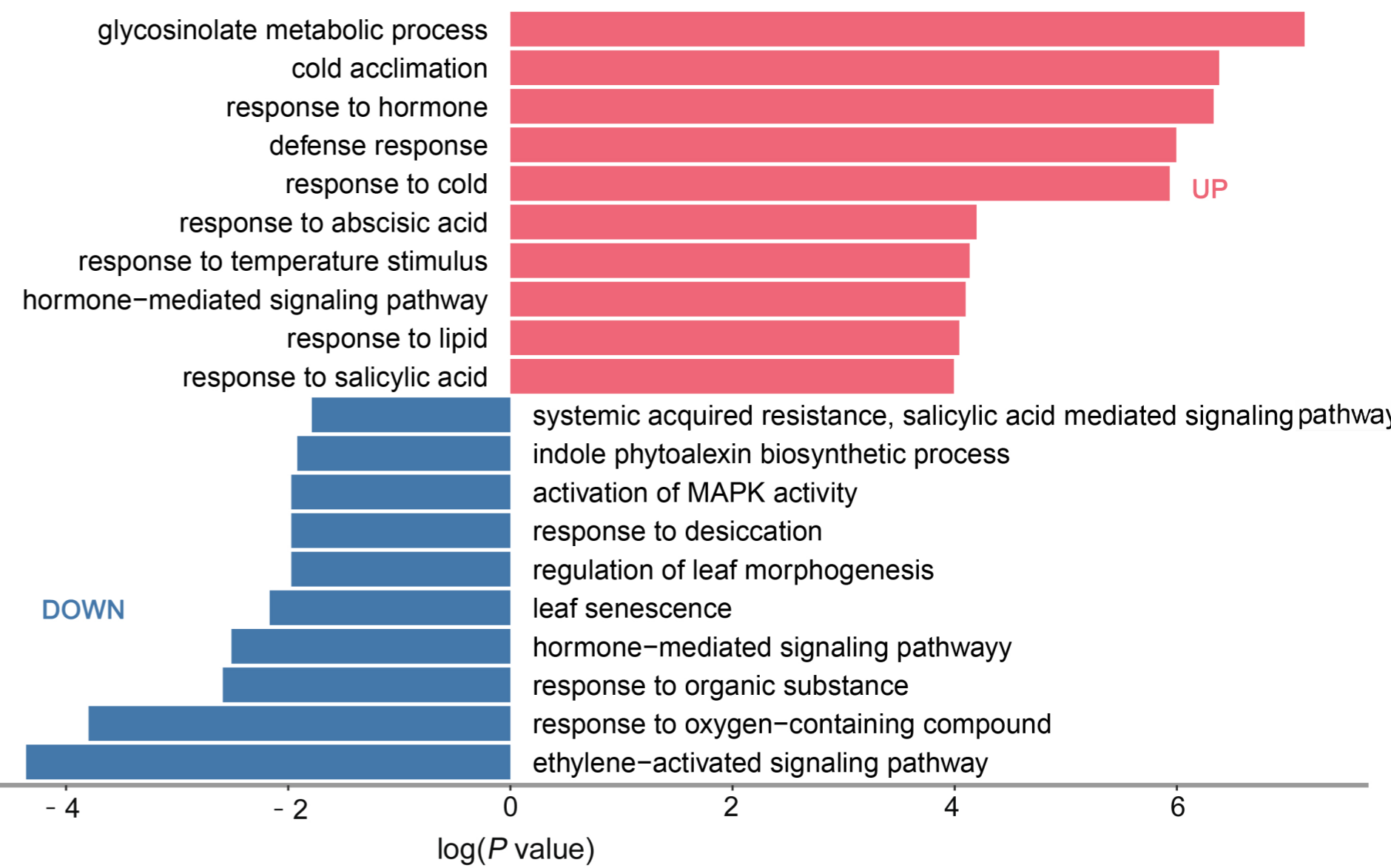

Recovery 2 d: AMHA vs. Mock

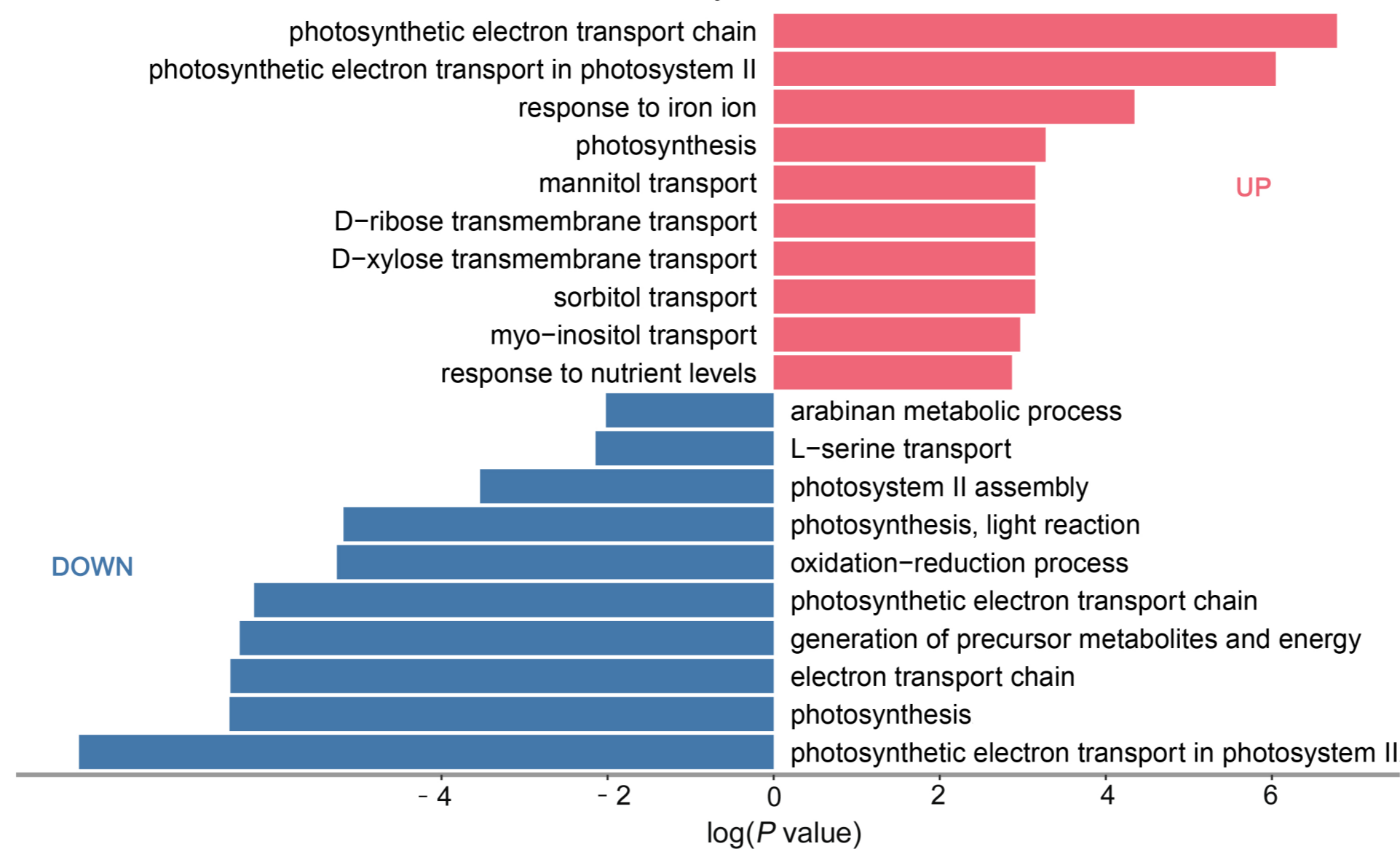

Supplement: Web_Material_uhaf073 [file web_material_uhaf073.zip › S5.pdf]

(a)

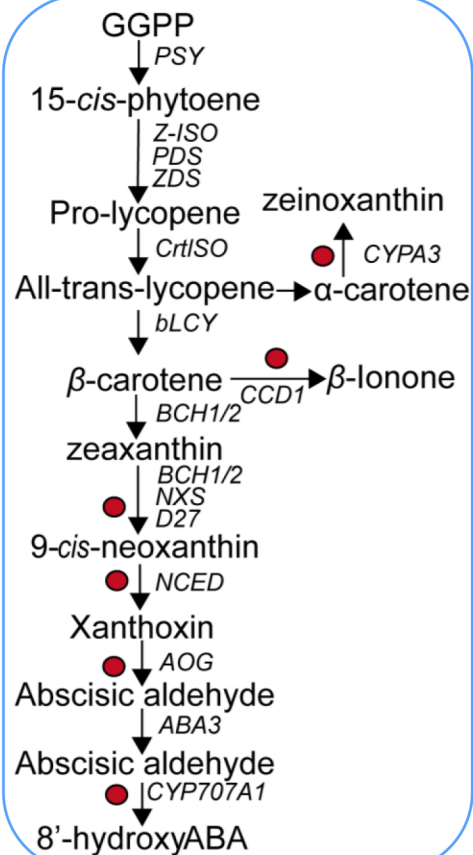

(b)

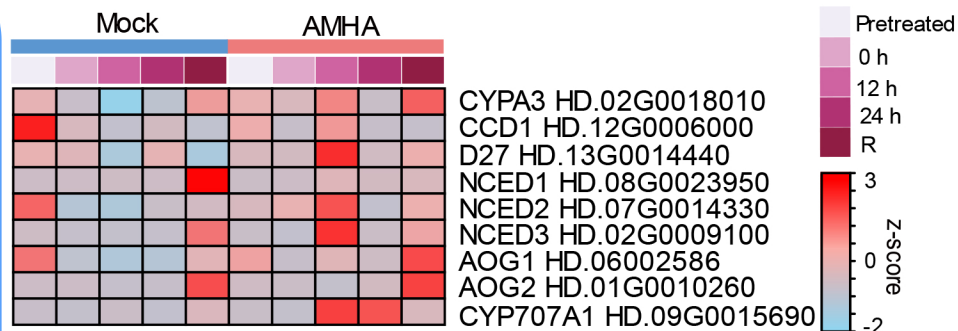

(c)

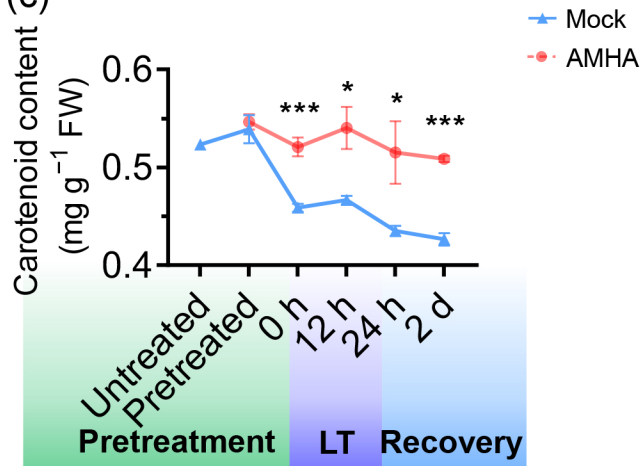

Supplement: Web_Material_uhaf073 [file web_material_uhaf073.zip › S6.pdf]

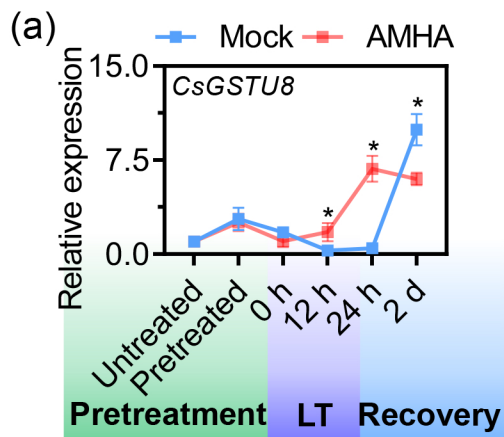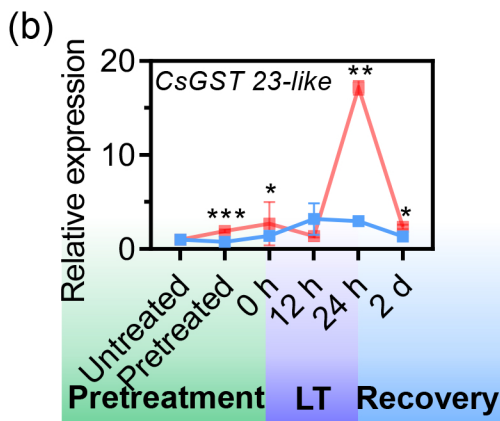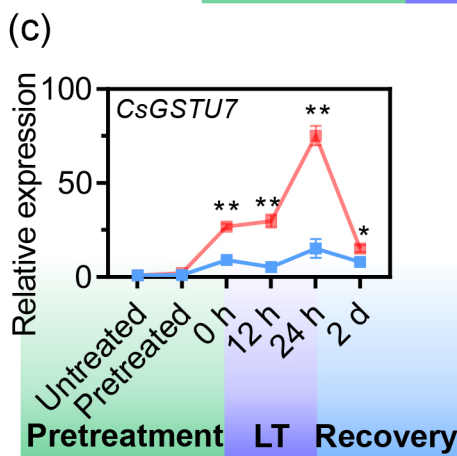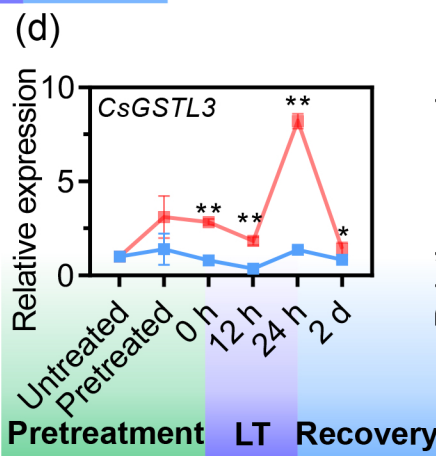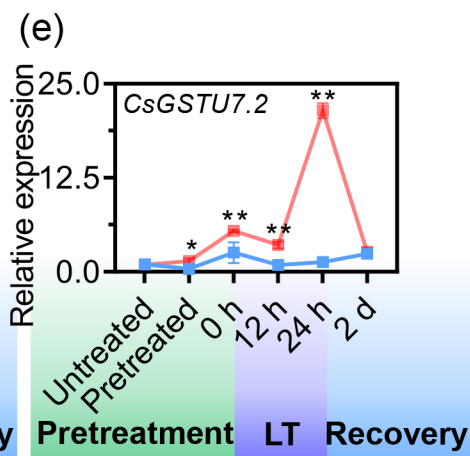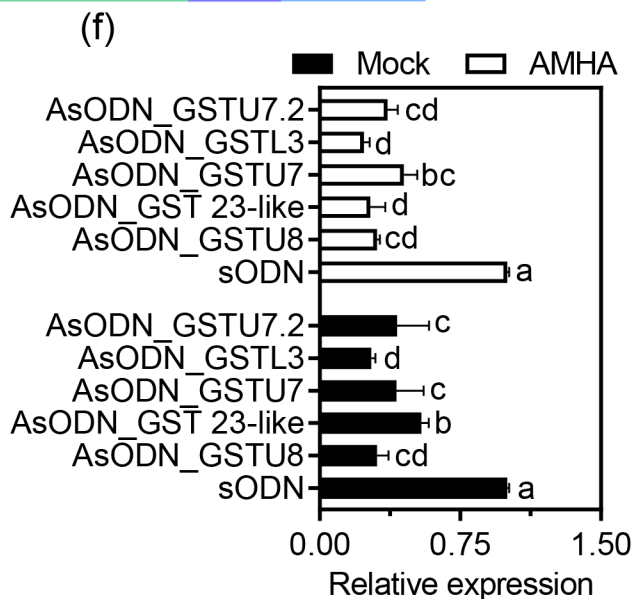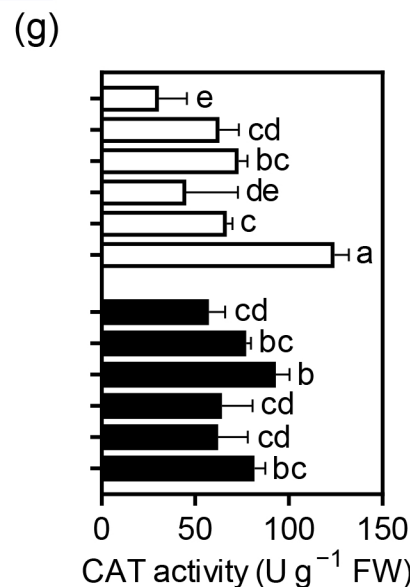

Supplement: Web_Material_uhaf073 [file web_material_uhaf073.zip › S8.pdf]

■ Mock □ AMHA

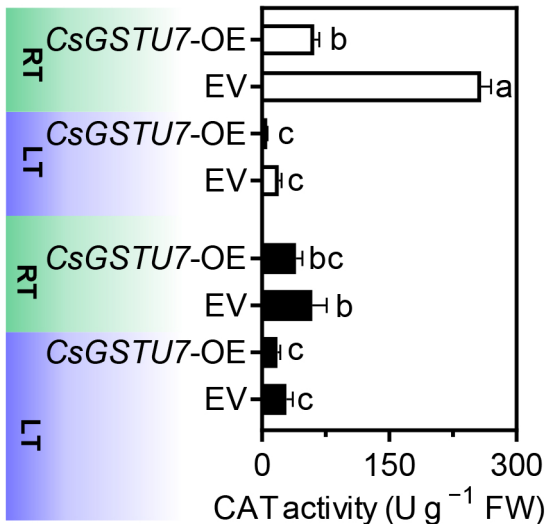

Supplement: Web_Material_uhaf073 [file web_material_uhaf073.zip › S9.pdf]
